# The functional role of m6A demethylase ALKBH5 in cardiomyocyte hypertrophy

**DOI:** 10.1038/s41419-024-07053-2

**Published:** 2024-09-18

**Authors:** Chen Meng, Haibi Su, Meiling Shu, Feng Shen, Yijie Lu, Shishi Wu, Zhenghua Su, Mengyao Yu, Di Yang

**Affiliations:** 1 Human Phenome Institute, Center for Medical Research and Innovation, Shanghai Pudong Hospital, Fudan University Pudong Medical Centre, Zhangjiang Fudan International Innovation Center, Shanghai Key Laboratory of Bioactive Small Molecules, Fudan University, Shanghai, China; 2 Department of Cardiovascular Surgery, Shanghai General Hospital, Shanghai Jiao Tong University of Medicine, Shanghai, China

**Keywords:** Heart failure, Methylation

## Abstract

Cardiomyocyte hypertrophy is a major outcome of pathological cardiac hypertrophy. The m6A demethylase ALKBH5 is reported to be associated with cardiovascular diseases, whereas the functional role of ALKBH5 in cardiomyocyte hypertrophy remains confused. We engineered *Alkbh5* siRNA (si*Alkbh5*) and *Alkbh5* overexpressing plasmid (*Alkbh5* OE) to transfect cardiomyocytes. Subsequently, RNA immunoprecipitation (RIP)-qPCR, MeRIP-qPCR analysis and the dual-luciferase reporter assays were applied to elucidate the regulatory mechanism of ALKBH5 on cardiomyocyte hypertrophy. Our study identified ALKBH5 as a new contributor of cardiomyocyte hypertrophy. ALKBH5 showed upregulation in both phenylephrine (PE)-induced cardiomyocyte hypertrophic responses in vitro and transverse aortic constriction (TAC)/high fat diet (HFD)-induced pathological cardiac hypertrophy in vivo. Knockdown or overexpression of ALKBH5 regulated the occurrence of hypertrophic responses, including the increased cardiomyocyte surface areas and elevation of the hypertrophic marker levels, such as brain natriuretic peptide (BNP) and atrial natriuretic peptide (ANP). Mechanically, we indicated that ALKBH5 activated JAK2/STAT3 signaling pathway and mediated m6A demethylation on *Stat3* mRNA, but not *Jak2* mRNA, resulting in the phosphorylation and nuclear translocation of STAT3, which enhances the transcription of hypertrophic genes (e.g., *Nppa*) and ultimately leads to the emergence of cardiomyocytes hypertrophic growth. Our work highlights the functional role of ALKBH5 in regulating the onset of cardiomyocyte hypertrophy and provides a potential target for hypertrophic heart diseases prevention and treatment.

**Figure:**
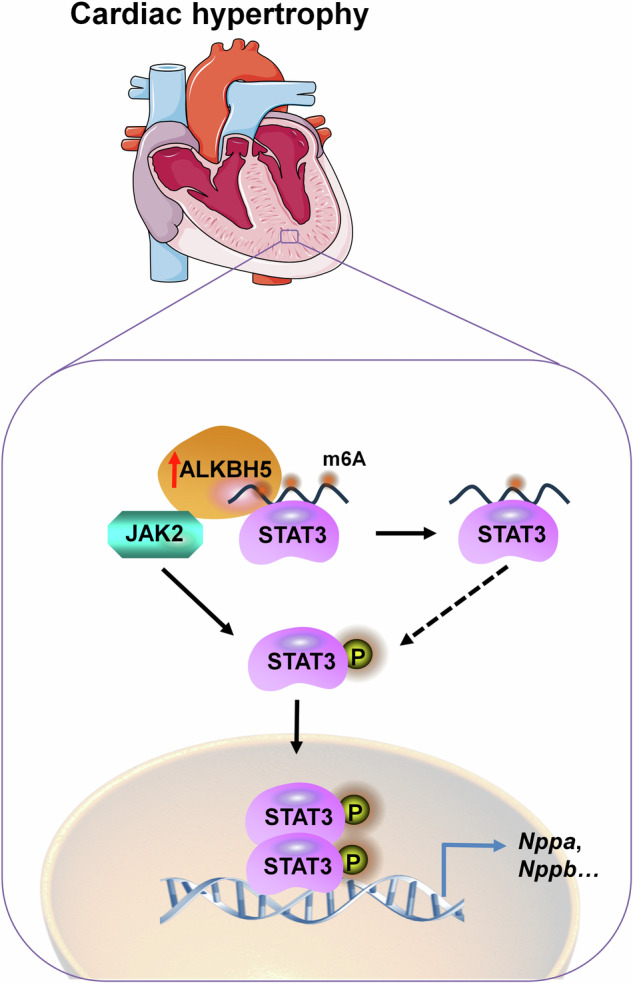
ALKBH5 activated JAK2/STAT3 signaling pathway and mediated m6A demethylation on *Stat3* mRNA, but not *Jak2* mRNA, resulting in the phosphorylation and nuclear translocation of STAT3, which enhances the transcription of hypertrophic genes (e.g., *Nppa*) and ultimately leads to the emergence of cardiomyocytes hypertrophic growth.

ALKBH5 activated JAK2/STAT3 signaling pathway and mediated m6A demethylation on *Stat3* mRNA, but not *Jak2* mRNA, resulting in the phosphorylation and nuclear translocation of STAT3, which enhances the transcription of hypertrophic genes (e.g., *Nppa*) and ultimately leads to the emergence of cardiomyocytes hypertrophic growth.

## Introduction

Pathological cardiac hypertrophy is regarded as an adverse outcome of hypertension, ultimately leading to heart failure (HF), the final stage of heart diseases with high morbidity and mortality, posing a serious threat to human life and health [1–3]. Therefore, delaying the onset of pathological cardiac hypertrophy is encouraging to prevent deterioration into HF [4].

The hallmarks of pathological cardiac hypertrophy include a growth in the surface area of cardiomyocytes, namely cardiomyocyte hypertrophy [5]. This condition also involves the reactivation of embryonic genes, such as increased expressions of atrial natriuretic peptide (ANP) and brain natriuretic peptide (BNP), as well as the transformation of contractile α-MHC subtypes (MYH6) into embryonal β-MHC subtypes (MYH7). Additionally, there is an increase in myocardial fibrosis, which eventually enlarged the reconstructed heart volume and reduced cardiac function [6, 7]. Despite extensive research, adequate targets for preventing the progression of pathological cardiac hypertrophy remain elusive. Discovering new intervention targets and molecular mechanisms to decrease the hypertrophic cardiomyocytes, are of great significance to improve cardiac function and long-term prognosis of HF patients.

N6-methyl-adenosine (m6A) is the most prevalent post-transcriptional modification in mammals and provides a new dimension to the prospect of gene post-transcriptional regulation [8]. m6A methylation plays a crucial role throughout the RNA life cycle, which affects the development of human diseases including cardiovascular diseases and cancers [9, 10]. AlkB homolog 5 (ALKBH5) is a m6A demethylase to remove the m6A modification of target RNAs, playing important regulatory functions in mRNA metabolism, transportation and stability [11]. Meanwhile, ALKBH5 is associated with various human diseases, including colon cancer [12], cerebral ischemia-reperfusion injury [13], *etc*. Importantly, aberrant expression of ALKBH5 has been implicated in the pathogenesis of various cardiovascular diseases. Notably, ALKBH5 deficiency promotes post-ischemic angiogenesis by stabilizing post-transcription of WNT5A [14]. Moreover, both ALKBH5 and m6A methyltransferase KIAA1429 are up-regulated in aortic tissues of patients with aortic dissection [15]. Furthermore, ALKBH5 and another prominent m6A methyltransferase METTL3 have been reported to reverse-regulate the m6A modification of the transcription factor TFEB mRNA, affecting the fate of hypoxia/reoxygenation (H/R)-treated cardiomyocytes [16]. Nevertheless, the functional role of ALKBH5 in cardiomyocyte hypertrophy remains unclear.

Numerous studies have shown the involvement of the janus kinase 2 (JAK2)-signal transducers and activators of transcription 3 (STAT3) pathway in the pathology of cardiac hypertrophy and HF [17]. STAT3 is regarded as the most important downstream factor of JAK2 in the initiation and development of cardiac hypertrophy. In response to external stimuli, STAT3 is phosphorylated by JAK2 at tyrosine 705 (Tyr705), leading to its translocation from the cytoplasm to the nucleus, which promotes the transcription of various hypertrophic factors, including natriuretic peptide type A (*Nppa*) and natriuretic peptide type B (*Nppb*) [18]. Moreover, it is noteworthy that cardiomyocyte-specific overexpressed STAT3 (Stat3-Tg) in mice results in spontaneous cardiac hypertrophy [19]. Interestingly, overexpression of the ALKBH5-HOXA10 loop can activate the JAK2/STAT3 signaling pathway by mediating JAK2 m6A demethylation and result in EOC chemoresistance [20]. However, the relationship between ALKBH5 and JAK2/STAT3 pathway in response to cardiomyocyte hypertrophy remains largely unknown.

In this study, we sought to investigate the role of ALKBH5 in regulating the onset of cardiomyocyte hypertrophic responses. We revealed that the mRNA and protein levels of ALKBH5 were upregulated in both phenylephrine (PE)-induced cardiomyocyte hypertrophic responses in vitro and TAC/high fat diet (HFD)-induced cardiac hypertrophy in vivo. Knockdown or overexpression of ALKBH5 regulated the occurrence of cardiomyocyte hypertrophic responses, including the augmented cardiomyocyte surface areas and upregulation of the hypertrophic marker levels. Mechanically, ALKBH5 activated JAK2/STAT3 signaling pathway and mediated m6A demethylation on *Stat3* mRNA, but not *Jak2* mRNA, which in turn promotes the phosphorylation of STAT3 to translocate into the nucleus, enhancing the transcription of hypertrophic genes, such as *Nppa*, ultimately leading to the development of cardiomyocyte hypertrophy.

## Results

### ALKBH5 is related to the occurrence of pathological cardiac hypertrophy

**Fig. 1:**
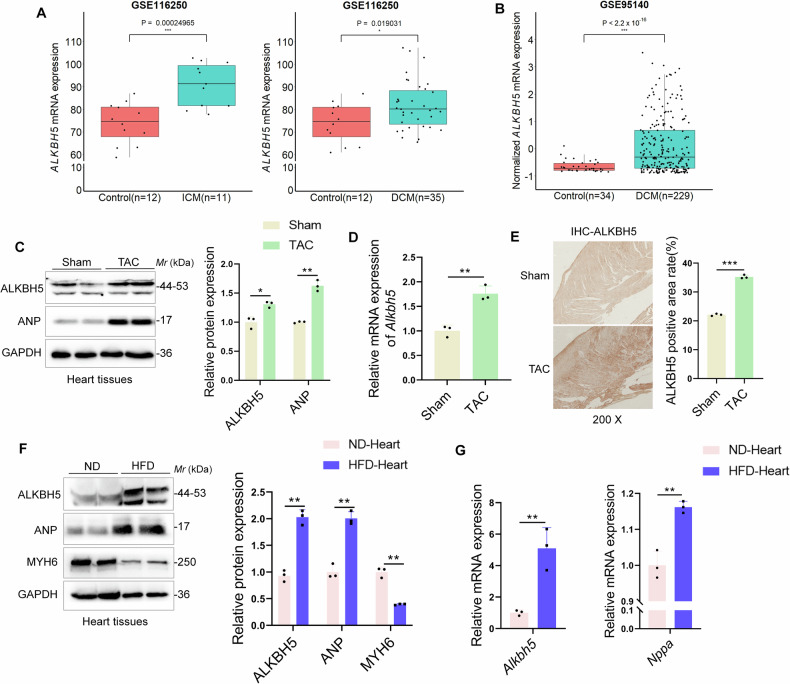
ALKBH5 is related to the occurrence of pathological cardiac hypertrophy. **A**, **B** The transcriptional profile of ALKBH5 was analyzed in the published human RNA-sequencing (RNA-seq) data (GSE116250) comprising with normal myocardium (Control, *n* = 12), patients with dilated cardiomyopathy (DCM, *n* = 35), and patients with ischemic cardiomyopathy (ICM, *n* = 11) and the published human RNA-seq data (GSE179107) including 229 human DCM samples and 34 controls. Gene expression analysis was conducted using the *t*-test method after filtering. *P* = 0.00024965 in ICM and *P* = 0.019031 in DCM, compared to the normal myocardium (Control) in GSE116250. *P* < 2.2 × 10^−16^ in DCM, compared to the normal myocardium (Control) in GSE179107. **C** ALKBH5 and ANP protein levels were detected by immunoblotting in the hearts of mice subjected to sham or TAC surgery (*n* = 3). **D** The qRT-PCR assay was performed to detect the mRNA level of *Alkbh5* gene in TAC-induced cardiac hypertrophy (*n* = 3). **E** Representative images and quantitation of immunohistochemical staining of ALKBH5 in the hearts of mice subjected to sham or TAC surgery. **F**, **G** Mice were fed a high fat diet (HFD) or normal diet (ND) for 12 weeks and the hearts were harvested. Western blots and qRT-PCR assays were performed to detect the protein and mRNA levels of ALKBH5, as well as hypertrophic biomarkers ANP and MYH6 protein or mRNA levels. *n* = 3 per group, data shown are means ± SD. ∗*p* < 0.05, ∗∗*p* < 0.01, ∗∗∗*p* < 0.001.

To define the potential role of ALKBH5 in pathological cardiac hypertrophy, we firstly analyzed the available data from human RNA-sequencing (RNA-seq) (GSE116250) [21, 22], namely transcriptomic data of 58 samples from human left ventricle tissue. We observed that the transcriptional profile of *ALKBH5* increased in left ventricle tissue of patients with ischemic cardiomyopathy (ICM) (*P* = 0.000242) or dilated cardiopathy (DCM) (*P* = 0.019) compared with donors with nonfailing (Control) myocardium (Fig. 1A). Moreover, we conducted a *t*-test on RNA-seq data obtained from the GSE95140 dataset [22], which includes 229 human DCM samples and 34 controls and found ALKBH5 was also highly expressed in DCM compared to controls (*P* < 2.2 × 10^−16^, log_2_FC = 1.79), providing further support for its role in DCM (Fig. 1B).

Next, we established a classical pathological cardiac hypertrophy mice model by transverse aortic constriction (TAC) surgery, which displayed impaired ventricular diastolic and dilatation function, and owned the consistent features with human dilated cardiomyopathy. Then we detected the protein expressions of ALKBH5 and the hypertrophic marker ANP in hearts of TAC-operated mice and found both ALKBH5 and ANP were shown to be more abundantly expressed in the hypertrophic hearts of mice (Fig. 1C), which the immunohistochemistry results corroborated in further detail (Fig. 1E). Moreover, the mRNA level of *Alkbh5* gene was also upregulated in TAC-induced cardiac hypertrophy (Fig. 1D), which is consistent with the transcriptional results from humans (Fig. 1A, B). Furthermore, it has been well documented that obesity significantly influences cardiac hypertrophy and dysfunction. To establish the specific contribution of ALKBH5 to cardiac hypertrophy and HF associated with metabolic syndrome, high-fat diets (HFD) were given to the mice to obtain the obesity-induced cardiac hypertrophy model. The protein and mRNA levels of the hypertrophic marker ANP were increased while MYH6 was decreased (Fig. 1F, G), demonstrating that HFD-induced cardiac hypertrophy model was successfully established. Surprisingly, both the protein and mRNA levels of ALKBH5 were upregulated in HFD-induced cardiac hypertrophy (Fig. 1F, G), while Fat mass and obesity-associated protein (FTO), the first reported m6A demethylase in eukaryotic cells, was also upregulated but not as high as ALKBH5 (Fig. S1). These above results showed that ALKBH5 is related to the occurrence of diverse pathological cardiac hypertrophy.

### ALKBH5 increases in response to cardiomyocyte hypertrophy in vitro

**Fig. 2:**
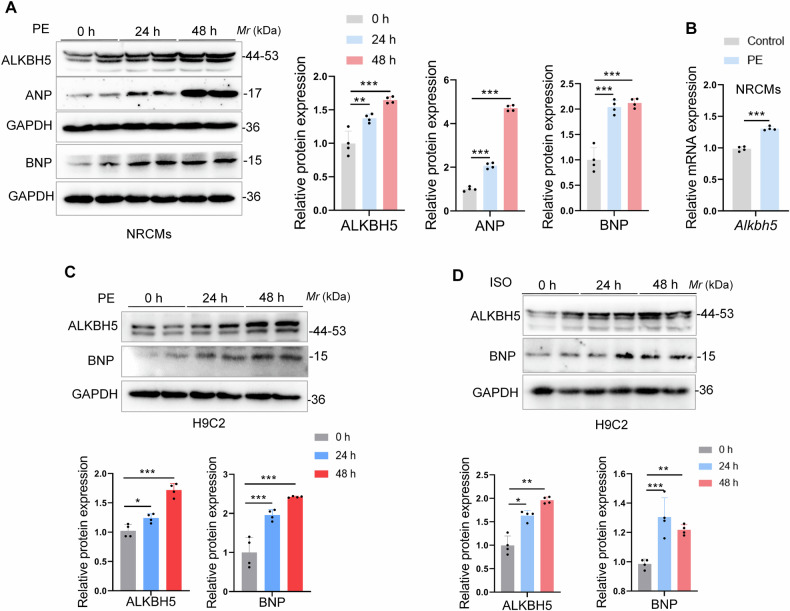
ALKBH5 increases in response to cardiomyocyte hypertrophy in vitro. **A** NRCMs were stimulated by phenylephrine (PE, 100 µM) to induce the hypertrophic responses in vitro and the protein levels of ALKBH5 and hypertrophic biomarkers ANP and BNP were examined by immunoblots. The quantifications were shown on the right. **B** The mRNA level of *Alkbh5* gene in PE (100 µM, 24 h)-induced NRCMs was detected by qRT-PCR. **C** The protein levels of ALKBH5 and BNP were examined in PE-induced H9C2 cells by western blots. **D** The protein expressions of ALKBH5 and BNP were examined in ISO (30 µM)-induced H9C2 cells by western blots. *n* = 4 per group. Data are expressed as the mean ± SD. ∗*p* < 0.05, ∗∗*p* < 0.01, ∗∗∗*p* < 0.001.

To investigate whether ALKBH5 regulates cardiomyocyte hypertrophy so as to result in cardiac hypertrophy in diverse pathological conditions, we examined ALKBH5 expressions in cardiomyocytes under classical hypertrophic agonists treatments, such as PE and isoprenaline (ISO). Firstly, the protein and mRNA levels of ALKBH5 were significantly increased in neonatal rat cardiomyocytes (NRCMs) under PE stimulation, which was in parallel with the upregulation of hypertrophic biomarkers ANP and BNP proteins (Fig. 2A, B). Similarly, this result was also confirmed in H9C2 cells (Fig. 2C). Furthermore, we also found that the expression of ALKBH5 was increased in ISO-induced H9C2 cells and NRCMs, accompanied by the upregulation of BNP (Figs. 2D and S2). These above data suggest that ALKBH5 is related to cardiomyocyte hypertrophy in vitro.

### Depletion of ALKBH5 alleviates PE-induced cardiomyocyte hypertrophy in vitro

**Fig. 3:**
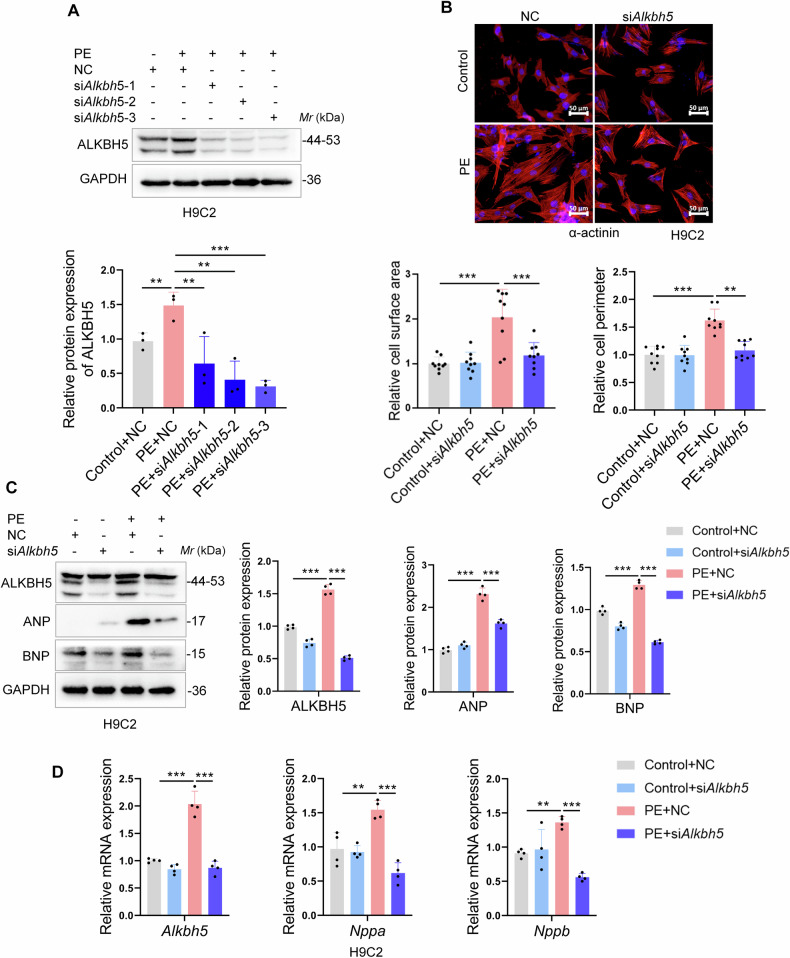
Depletion of ALKBH5 alleviates PE-induced cardiomyocyte hypertrophy in vitro. H9C2 cells were transfected with si*Alkbh5* or NC and then treated with PE (100 µM) for 48 h. **A** The depletion efficiency of three si*Alkbh5s* were tested by western blots and the quantification was shown on the right. **B** Representative immunofluorescence images (left) and quantification (right, the relative cell surface area and cell perimeter) of α-actinin staining in PE-triggered H9C2 cells. Scale bar, 50 μm. **C**, **D** Relative protein and mRNA expressions of ALKBH5, ANP and BNP in H9C2 cells treated with si*Alkbh5* or NC and then stimulated with PE (100 µM) for 48 h (*n* = 4 per group). Data are expressed as the mean ± SD. ∗*p* < 0.05, ∗∗*p* < 0.01, ∗∗∗*p* < 0.001.

To directly monitor the functional role of ALKBH5 in cardiomyocyte hypertrophy, we introduced small interfering RNAs (siRNAs) targeting ALKBH5 (si*Alkbh5*) or NC (the negative control) to transfect H9C2 cells or NRCMs. Firstly, the efficiency of three si*Alkbh5s* were tested in both H9C2 cells and NRCMs and all the si*Alkbh5s* markedly prevented the increase of the ALKBH5 induced by PE but si*Alkbh5*-3 was the most efficient one, which was selected for the following study (Figs. 3A and S3A). Then the immunofluorescence staining of α-actinin, a cytoskeletal protein, was performed to measure the cardiomyocyte surface areas. The results indicated that depletion of ALKBH5 with si*Alkbh5* showed lower cardiomyocyte surface area in PE-induced H9C2 cells or NRCMs (Figs. 3B and S3B). Of note, both the upregulated protein and mRNA levels of PE-induced ANP and BNP were further decreased by si*Alkbh5* treatment in H9C2 cells (Fig. 3C, D). Similar to the pattern in H9C2 cells, the hypertrophic markers ANP and BNP, and the hypertrophy related transcription factor GATA-binding protein 4 (GATA4), an important member of the GATA family of transcription factors and has been confirmed to play an important role in the process of cardiac hypertrophy [23], were also downregulated with si*Alkbh5* transfection in PE-induced NRCMs (Fig. S3C). Collectively, these above findings demonstrated that depletion of ALKBH5 alleviates PE-induced cardiomyocyte hypertrophy.

### ALKBH5 over-expression promotes cardiomyocyte hypertrophic responses

**Fig. 4:**
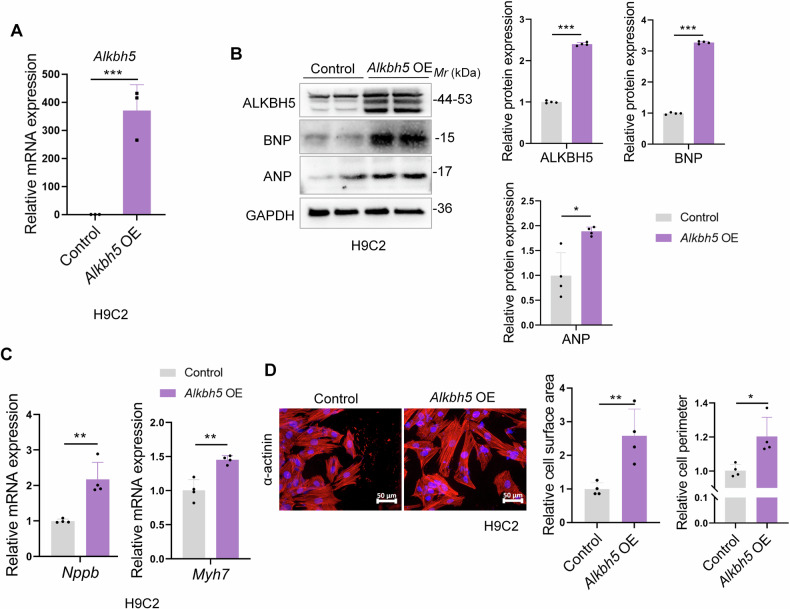
ALKBH5 over-expression promotes cardiomyocyte hypertrophic responses. H9C2 cells were transfected with *Alkbh5* OE or vector. **A** The efficiency of *Alkbh5* OE plasmid were tested by qRT-PCR. **B** Representative immunoblots of hypertrophic markers ANP and BNP upon *Alkbh5* OE transfection. **C** qRT-PCR assays were performed to test the hypertrophic marker gene *Nppb* and *Myh7* (*n* = 4 per group). **D** The immunofluorescence images (left) and quantification (right, the relative cell surface area and cell perimeter) of α-actinin staining in *Alkbh5* OE-triggered H9C2 cells. Data are expressed as the mean ± SD. ∗*p* < 0.05, ∗∗*p* < 0.01, ∗∗∗*p* < 0.001.

To explore the outcomes of excessive ALKBH5 expression in cardiomyocytes, we transduced H9C2 cells with flag-tagged ALKBH5 overexpressed plasmids (*Alkbh5* OE), along with a lentiviral vector as the control. Firstly, the overexpression efficiency of ALKBH5 plasmid transfecting H9C2 cells was verified in both mRNA and protein levels (Fig. 4A, B). Then we further found that the protein expressions of hypertrophic markers ANP and BNP were increased in both *Alkbh5* OE-induced H9C2 cells and NRCMs (Figs. 4B and S4A). Besides, the mRNA levels of the hypertrophic marker *Nppb* and *Myh7* genes were also upregulated in *Alkbh5* OE-induced H9C2 cells (Fig. 4C). In addition, the hypertrophy related transcription factor GATA4 was also upregulated in *Alkbh5* OE-induced H9C2 cells (Fig. S4C). Furthermore, nuclear factor of activated T cells 4 (*Nfatc4*) and myocyte enhancer factor 2C (*Mef2c*), two prohypertrophic transcription factors that promoted the expression of hypertrophic genes [24, 25], were also increased *Alkbh5* OE-induced cardiomyocyte hypertrophy (Fig. S4B, D). Take a further step, the augmented cardiomyocyte surface areas and cell perimeters were also observed in response to *Alkbh5* OE treatment in both NRCMs and H9C2 cells (Figs. 4C and S4E). Altogether, the aforementioned findings showed that overexpression of ALKBH5 directly induced hypertrophic responses, which is necessary for the advancement of cardiomyocyte hypertrophic development in vitro.

### ALKBH5 activated JAK2/STAT3 pathway and triggered STAT3 phosphorylation and nuclear translocation

**Fig. 5:**
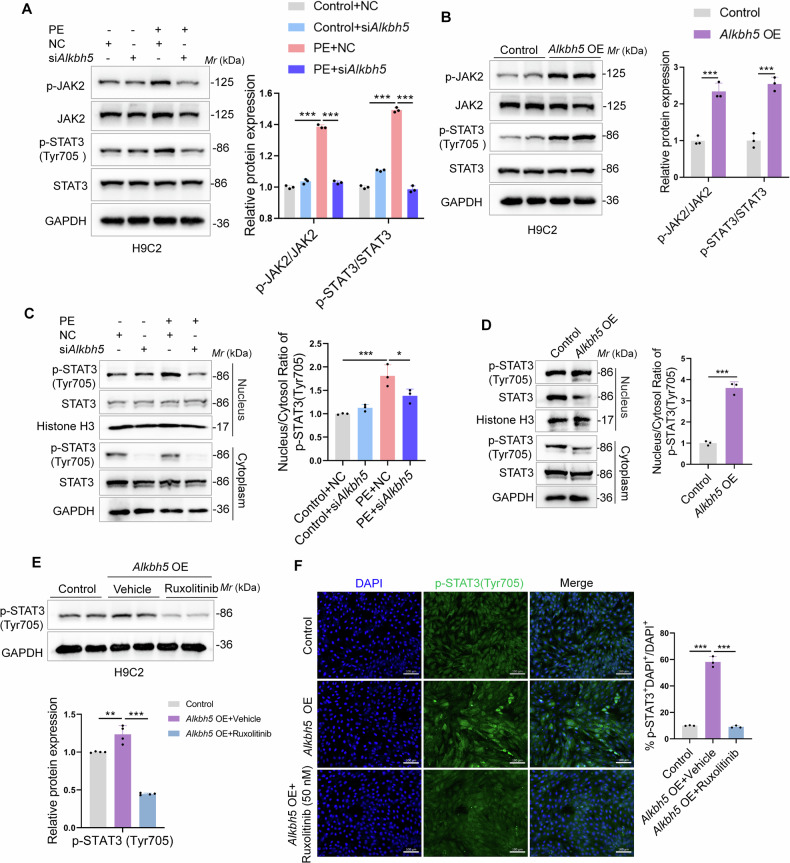
ALKBH5 activated JAK2/STAT3 pathway and triggered STAT3 phosphorylation and nuclear translocation. **A**, **B** H9C2 cells were transfected with *Alkbh5* OE alone or si*Alkbh5* upon PE treatment. Immunoblot analysis were performed to detect the levels of p-JAK2 and p-STAT3(Tyr705), as well as the total protein expressions of JAK2 and STAT3. **C**, **D** H9C2 cells were transfected with si*Alkbh5* upon PE treatment or *Alkbh5* OE alone. The nucleus or cytoplasmic fraction of H9C2 cells was lysis with RIPA, followed by subjection to western blot analysis. The quantification referred to the ratio of nuclear p-STAT3/STAT3 to cytoplasmic p-STAT3/STAT3 (*n* = 3 for each group) were shown on the right. **E** The JAK2 inhibitor Ruxolitinib was employed for western blot assays to examine the expression of p-STAT3 (Tyr705) in *Alkbh5* OE-induced H9C2 cells. **F** Immunofluorescence staining of p-STAT3(Tyr705) indicated the subcellular location of p-STAT3(Tyr705) in H9C2 cells treated with *Alkbh5* OE transfection with or without Ruxolitinib treatment in H9C2 cells. The quantification by calculating the ratio of the cells with nuclear-positive staining for p-STAT3 was shown on the right panel. Scale bar, 100 μm. Data are expressed as the mean ± SD. ∗∗*p* < 0.01, ∗∗∗*p* < 0.001.

Numerous studies have shown that the JAK2/STAT3 pathway participates in the pathology of cardiac hypertrophy and HF [17]. Thus, to explore the downstream regulator of ALKBH5 in cardiomyocyte hypertrophy, the JAK2/STAT3 pathway piqued our interest due to its pivotal role in cardiac hypertrophy [26]. We sought to determine whether ALKBH5 modulated JAK2/STAT3 pathway so as to regulate the cardiomyocyte hypertrophic growth. As seen in the Fig. 5A, B, the JAK2/STAT3 pathway was directly activated by *Alkbh5* OE, whereas was eliminated upon ALKBH5 knockdown in PE-induced H9C2 cells. Similar results were obtained in NRCMs (Fig. S5A, B). Since the phosphorylation and nuclear translocation of STAT3 promoted the transcription of hypertrophic genes [21], we examined the regulatory functions of ALKBH5 on STAT3. As seen in Fig. 5C, we confirmed that STAT3 nuclear translocation was increased upon PE treatment, which was inhibited by *Alkbh5* siRNA in PE-induced H9C2 cells by measuring p-STAT3 levels of both nuclear and cytoplasm. Similarly, the p-STAT3 nuclear translocation was also increased upon *Alkbh5* OE treatment (Fig. 5D). Additionally, the immunofluorescence staining was performed to visualize the nuclear translocation of p-STAT3 in *Alkbh5* OE transfected-H9C2 cells (Fig. 5F) as well as in PE-induced H9C2 cells, which was inhibited upon si*Alkbh5* (Fig. S5C). To further elaborate whether ALKBH5-induced STAT3 phosphorylation and nuclear translocation is dependent of JAK2, we employed the JAK2 inhibitor, Ruxolitinib and conducted the western blot and immunofluorescent staining assays. The results indicated that p-STAT3 (Tyr705) was significantly decreased in *Alkbh5* OE-induced H9C2 cells (Fig. 5E) and the translocation to the nucleus is also reduced (Fig. 5F). These data indicated that ALKBH5 activated JAK2/STAT3 pathway and triggered STAT3 phosphorylation and nuclear translocation via JAK2.

### ALKBH5 activated STAT3 in a m6A-dependent manner

**Fig. 6:**
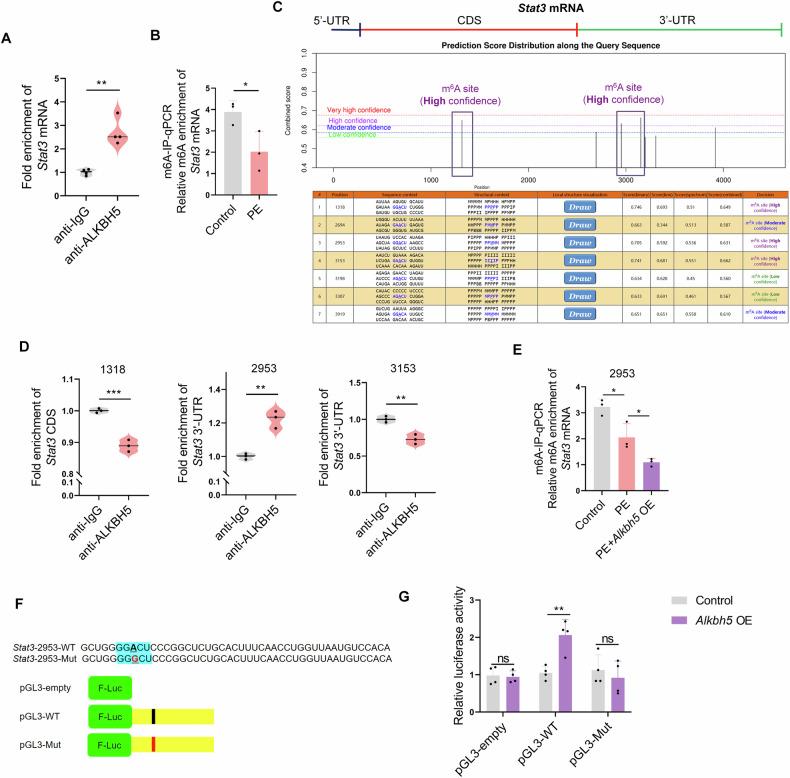
ALKBH5 regulates STAT3 activity in a m6A-dependent manner. **A** Relative enrichment of *Stat3* mRNA associated with ALKBH5 protein was identified by RIP-qPCR assays using anti-ALKBH5 antibody. *n* = 4. **B** MeRIP-qPCR assays were performed to detect the relative m6A enrichment of *Stat3* mRNA in PE-induced H9C2 cells. *n* = 4. **C** The online prediction of *Stat3* mRNA in SRAMP website showed the potential sites of m6A modification. The purple boxes represent m6A sites with high confidence. **D** RIP-qPCR analysis of the enrichment of ALKBH5 protein on predicted m6A modification sites of *Stat3* mRNA CDS and 3’-UTR regions (*Stat3* CDS-1318, *Stat3* 3’-UTR-2953, *Stat3* 3’-UTR-3153) in H9C2 cells. **E** m6A modification of *Stat3* 3’-UTR-2953 was detected by MeRIP-qPCR analysis using m6A antibody in *Alkbh5* OE-transfected H9C2 cells upon PE treatment. **F** The mutation at the putative m6A site (2953) in *Stat3* 3’-UTR (A to G) was generated and the *Stat3* mRNA coding sequence (pGL3-WT) and *Stat3* 3’-UTR-2953 mutated sequence (pGL3-Mut) luciferase reporter vectors were constructed. **G** Relative luciferase activity of the pGL3-empty, pGL3-WT, and pGL-Mut luciferase reporter in Control and *Alkbh5* OE-induced H9C2 cells. Data are expressed as the mean ± SD. ∗*p* < 0.05, ∗∗*p* < 0.01, ∗∗∗*p* < 0.001.

Since ALKBH5 is a m6A demethylase, we next investigated whether ALKBH5 activated JAK2/STAT3 pathway in a m6A-dependent manner. Firstly, we performed RIP-qPCR assays to detect the interactions between ALKBH5 and JAK2/STAT3 pathway. Interestingly, ALKBH5 could bind with both *Jak2* and *Stat3* mRNA (Figs. 6A and S6A). To determine whether JAK2 or STAT3 is the direct downstream effector of m6A demethylation by ALKBH5 in PE-induced cardiomyocyte hypertrophy, we performed MeRIP-qPCR assays, which utilize immunoprecipitation to obtain RNA fragments enriched with m6A modifications, followed by quantitative PCR to measure their abundance, elucidating the distribution and regulation of m6A modifications in RNA transcripts. The results showed that the relative m6A enrichment of *Jak2* mRNA did not change in PE-induced H9C2 cells or even increased in *Alkbh5* OE transfected-H9C2 cells (Fig. S6B, C). However, the relative m6A enrichment of *Stat3* mRNA was remarkably decreased in PE-induced H9C2 cells (Fig. 6B). Thus, we next focused on the effects of ALKBH5 on *Stat3* mRNA and screened the possible m6A modification sites in *Stat3* mRNA by SRAMP (http://www.cuilab.cn/sramp), a powerful online prediction tool to predict the m6A site on genes. As shown in Fig. 6C, there were three potential and high possibility m6A modification sites in *Stat3* mRNA CDS and 3’-UTR regions, namely position *Stat3* CDS-1318, *Stat3* 3’-UTR-2953, *Stat3* 3’-UTR-3153. Then we designed the primers that contain these positions and performed the RIP-qPCR assays to detect which position ALKBH5 was binding to. The results showed that ALKBH5 could bind with the *Stat3* 3’-UTR-2953, but not the *Stat3* CDS-1318 and *Stat3* 3’-UTR-3153 (Fig. 6D). Next, we performed MeRIP-qPCR assays and found remarkable decreases of m6A modification at *Stat3* 3’-UTR-2953 in *Alkbh5* OE transfected-H9C2 cells upon PE treatment (Fig. 6E). In addition, we performed the mutation at *Stat3* 3’-UTR-2953 (A to G) and constructed the *Stat3* mRNA coding sequence (pGL3-WT) and *Stat3* 3’-UTR-2953 mutated sequence (pGL3-Mut) luciferase reporter vectors to test whether *Stat3* 3’-UTR-2953 mediated the m6A modification (Fig. 6F). The results showed that ALKBH5 overexpression increased the luciferase activity of pGL3-WT, but did not affect pGL3-Mut (Fig. 6G), indicating that the potential m6A site (*Stat3* 3’-UTR-2953) on *Stat3* mRNA is an ALKBH5-mediated demethylation site. Collectively, the above data demonstrated that ALKBH5 activated STAT3 in a m6A-dependent manner, particularly ALKBH5 demethylate *Stat3* mRNA at *Stat3* 3’-UTR-2953.

### ALKBH5 mediates cardiomyocyte hypertrophic growth via STAT3 activation

**Fig. 7:**
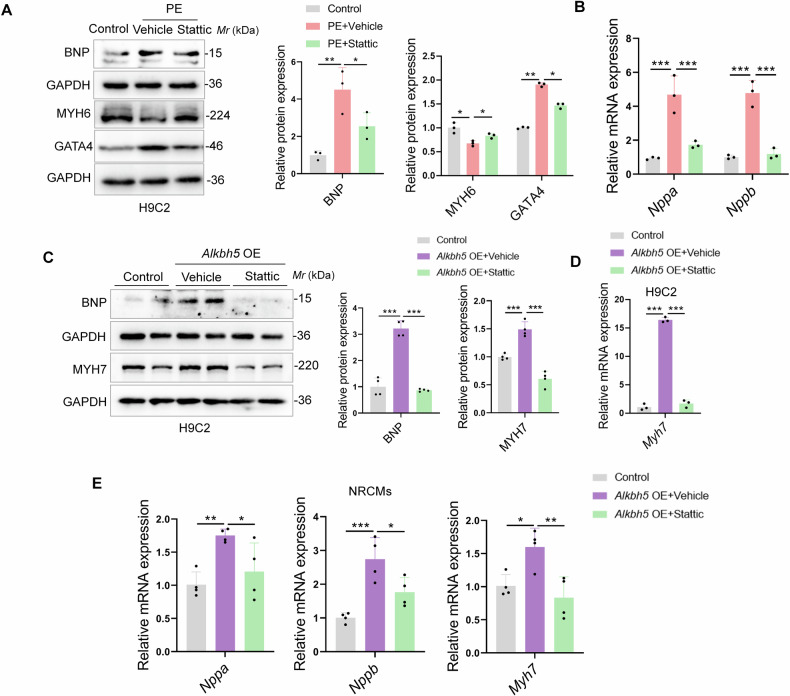
ALKBH5 mediates cardiomyocyte hypertrophic growth via STAT3 activation. **A**, **B** H9C2 cells were stimulated with PE, and the small-molecule inhibitor of STAT3, Stattic (10 µM) was added 4 h prior to PE treatment. Western blots and qRT-PCR were performed to detect the expressions of hypertrophic markers. **C**, **D** H9C2 cells were transfected with *Alkbh5* OE plasmid, and Stattic (10 µM) was added 4 h prior to *Alkbh5* OE transfection. Western blots and qRT-PCR were performed to detect the expressions of hypertrophic markers. **E** NRCMs were transfected with *Alkbh5* OE plasmid, and Stattic (10 µM) was added 4 h prior to *Alkbh5* OE transfection. qRT-PCR assays were performed to detect the expressions of hypertrophic markers. Data are expressed as the mean ± SD. ∗*p* < 0.05, ∗∗*p* < 0.01, ∗∗∗*p* < 0.001.

In order to explore whether STAT3 activation is necessary for initiating the transcription of downstream hypertrophic markers in PE or *Alkbh5* OE-induced H9C2 cells, we used the STAT3 small-molecule inhibitor, Stattic (10 µM), which has been reported to selectively inhibit dimerization and activation of STAT3 [27]. As seen in Fig. 7A, the hypertrophic marker BNP and GATA4 were induced while MYH6 was decreased upon PE stimuli, but reversed by Stattic treatment. And *Nppa* and *Nppb* genes were also downregulated upon Stattic treatment in PE-induced H9C2 cells (Fig. 7B). The similar results were also found in *Alkbh5* OE-induced H9C2 cells. Namely, the protein expressions of BNP and MYH7 were increased upon *Alkbh5* OE transfection, but reversed by Stattic treatment in H9C2 cells (Fig. 7C). In addition, qRT-PCR results showed *Nppa*, *Nppb*, and *Myh7* genes were also downregulated upon Stattic treatment in *Alkbh5* OE-induced H9C2 cells and NRCMs (Fig. 7D, E). The findings mentioned above indicate that ALKBH5 mediated cardiomyocyte hypertrophic growth via STAT3 activation.

## Discussion

In the present study, we pinpointed ALKBH5 as a key trigger to mediate the development of cardiomyocyte hypertrophic growth. In both NRCMs and H9C2 cells, overexpression of ALKBH5 increased whereas knockdown of ALKBH5 decreased PE-induced cardiomyocyte size growth and expressions of hypertrophy marker genes, such as *Nppa* and *Nppb*. Mechanically, ALKBH5 activated JAK2/STAT3 signaling pathway and mediated m6A demethylation on *Stat3* mRNA, but not *Jak2* mRNA, resulting in the phosphorylation and nuclear translocation of STAT3, which enhances the transcription of hypertrophic genes (e.g., *Nppa*) and ultimately leads to the emergence of hypertrophic cardiomyocytes. Harnessing ALKBH5 in cardiomyocytes might be an effective approach to break the progression of pathological cardiac hypertrophy.

The pathological growth of cardiomyocytes (cardiomyocyte hypertrophy) is a prominent feature during the development of cardiac hypertrophy [28]. Initially, the pressure overload to the heart is compensatory. If sustained, e.g., in hypertension, the compensated hypertrophied cardiomyocytes will transit to decompensated failing status [29]. Various signaling cascades are initially activated in response to the adaptive morphological hypertrophy during this process, subsequently transition to the failing trait, which is marked by contractile dysfunction and deterioration of cardiac performance [30]. Just as we previously reported [5], TAC-induced cardiac hypertrophy is a classical mice model, which is characterized by the increased heart weight/tibia length (HW/TL) ratios, downregulation of left ventricular (LV) ejection fraction (EF%) and LV fractional shortening (FS %), the increased interstitial fibrosis and cross-sectional diameter of cardiomyocytes, *etc*. Therefore, inhibition of cardiomyocyte hypertrophic growth is one of the strategies to prevent pathological cardiac hypertrophy. Consistent with these findings, we found that ALKBH5 could control the enlargement of cardiomyocytes upon PE treatment, which was measured by α-actinin staining. Namely, cardiomyocytes transfected with *Alkbh5* OE enlarged the cardiomyocyte surface areas while cardiomyocytes transfected with si*Alkbh5* showed lower cardiomyocyte surface areas, suggesting that ALKBH5 regulate cardiomyocyte hypertrophy.

As known, catecholamines mainly act on adrenergic receptors and excess catecholamines cause cardiac hypertrophy due to the reduced contractile responses to adrenergic agonists [31]. Studies have reported that there are a variety of adrenergic receptors in cardiomyocytes, including α1-adrenergic receptors (α1A, α1B and α1D) and β-adrenergic receptors (β1, β2 and β3) [32]. Phenylephrine (PE) is reported to activate α1 adrenergic receptor and also has a weak β receptor effect, which is regarded as an ideal cardiomyocyte hypertrophy stimulator [33]. ISO, a nonselective β-adrenergic receptor (β-AR) agonist, has been widely used as another stimulus for cardiac hypertrophy [34]. In our study, H9C2 cells or NRCMs were treated with 100 μM PE or 30 μM ISO, resulting in significant cardiomyocyte hypertrophy as indicated by the increased hypertrophic marker (BNP). Thus in the above successfully established cardiomyocyte hypertrophy model, we continued to examine the association between ALKBH5 and cardiomyocyte hypertrophy and found ALKBH5 significantly increases in response to PE or ISO-induced cardiomyocyte hypertrophy in vitro.

Furthermore, most studies of cardiac hypertrophy have used neonatal rat heart for primary culture of cardiomyocytes. However, the main disadvantage is the need for large numbers of neonatal rats. Given the increasing recognition of minimizing the number of animals in research, the use of cardiomyocyte lines, at least in some of these experimental researches, will be able to compensate for this disadvantage. It has been reported that almost identical hypertrophic responses of H9C2 cells were observed with those of NRCMs following hypertrophic agonists, such as angiotensin II or endothelin-1 [29]. It is now clear that H9C2 cells, a subclone of the original clonal cell line derived from embryonic BD1X rat heart tissue that exhibits many of the properties of skeletal muscle [35], could no longer beat, but they still share many similarities with NRCMs including membrane morphology and electrophysiological properties and can be used in cardiovascular disease research [36]. Keeping with these findings, the effect and mechanism of ALKBH5 in PE/ISO-induced cardiomyocyte hypertrophic responses were mainly validated in H9C2 cells, and only a portion of these hypertrophic phenotypes were synchronized to be validated in NRCMs as well.

Subsequent studies identified aberrant ALKBH5 expression plays a vital part in the pathogenesis of several cardiovascular diseases, such as aortic dissection, and post-ischemic angiogenesis [14, 15]. A recent study showed the decreased m6A level in Ang II-induced H9C2 cells hypertrophy, which is caused by the upregulation of ALKBH5 [37], indicating the potential function of ALKBH5 during the cardiac hypertrophy. Our study confirmed this hypothesis by indicating that ALKBH5 was profoundly increased in PE/ISO (another two different hypertrophic stimulants)-induced NRCMs and H9C2 cells. siRNA-mediated ablation of ALKBH5 endows NRCMs and H9C2 cells with resistance to cardiomyocyte hypertrophic responses, whereas overexpression of ALKBH5 using ALKBH5 lentiviral plasmids (*Alkbh5* OE) directly triggered cardiomyocyte hypertrophy, demonstrating that ALKBH5 indeed plays a functional role in cardiomyocyte hypertrophy.

The notable effects of ALKBH5 on cardiomyocyte hypertrophy motivated us to investigate the underlying mechanisms. Multiple potential ALKBH5 binding proteins have been reported in a set of documented studies. Among them, STAT3, one of the subtypes of STAT family, piqued our interest as it has been implicated to be constitutively activated in response to various hypertrophic stress [26, 38]. Mice that overexpressed STAT3 specifically in cardiomyocytes causes spontaneous concentric cardiac hypertrophy [19]. Upon the stimulus of proinflammatory cytokines *etc*., STAT3(Tyr705) is phosphorylated by receptor associated JAK2, then forms a homodimer and translocates to the nucleus where it binds to the promoter region and activates the transcription of target genes. Unlike the classic pathway, we found that STAT3, rather than JAK2, acted as a new m6A demethylation downstream factor of ALKBH5. Namely, ALKBH5 activated JAK2/STAT3 signaling pathway and mediated m6A demethylation on *Stat3* mRNA, but not *Jak2* mRNA, resulting in the phosphorylation and nuclear translocation of STAT3, which enhances the transcription of hypertrophic genes (e.g., *Nppa*) and ultimately leads to the emergence of cardiomyocytes hypertrophic growth.

Because of the limited experimental conditions, the in vivo animal experiments of ALKBH5 knockout or knock-in mice under TAC operation were not conducted, which will be studied in the future. Besides, the precise mechanism of ALKBH5-mediated m6A modification on STAT3 mRNA stability was not clearly articulated, such as the involved m6A readers, which should be illustrated in the following studies.

## Materials and methods

The data, methods and materials related to this study are available to other researchers upon reasonable request.

### Reagents

Reagent sources were as follows: phenylephrine (PE, S224411) was purchased from Yuanye Bio-Technology. The JAK2 inhibitor Ruxolitinib (S1378) was purchased from Selleckchem. Antibodies were obtained from the following commercial sources: ANP (Abcam, ab225844), BNP (Servicebio, GB11667), GAPDH (Proteintech, 60004-1-Ig), GATA4 (Santa Cruz Biotechnology, sc-25310), α-actinin (Santa Cruz Biotechnology, sc-166524), ALKBH5 (Proteintech, 16837-1-AP), m6A (Synaptic Systems, 202003), p-JAK2 (Cell Signaling Technology, C80C3), JAK2 (Cell Signaling Technology, 3230), p-STAT3 (Tyr705) (Cell Signaling Technology, 9145), STAT3 (Cell Signaling Technology, 9139), Histone H3 (Cell Signaling Technology, 4499).

### Animal studies

All the animal experimental procedures were conformed to the Animal Welfare Act Guide for Use and Care of Laboratory Animals, and an Institutional Animal Care and Use Committee (IACUC)-approved protocol of Fudan University, China. Mice were housed under specific pathogen-free (SPF) conditions at a stable temperature of 24 °C and fed with chow diet and water at libitum. The mice were randomly divided and the investigator was blinded to the group allocation of the mice during the experiment. The male mice were chosen due to the variables (uncertain hormone changes et al.) in female mice. The investigator was blinded to the group allocation of the mice during the experiment. The sample size is described in the corresponding figure legend. No animals were excluded from the analysis.

For the establishment of pathological cardiac hypertrophy mouse models, we employed a mouse model of cardiac hypertrophy: transverse aortic constriction (TAC). Firstly, C57BL/6J mice (24–26 g, male) were randomly selected and anesthetized with isoflurane using a small animal anesthesia machine (Shenzhen RWD Life Science Co., Ltd, Shenzhen, China). The induction concentration of anesthesia was 3% and the maintenance concentration was 1.5% throughout the surgery. The mice further intubated and connected to a volume-cycle rodent ventilator on supplemental oxygen and isoflurane at a rate of 1.0 L/min and respiratory rate of 140 bpm/min throughout the surgery. Next, thoracotomy was performed between the first and second rib and the thymus was pushed aside. The aortic arch was narrowed between the right innominate and left common carotid arteries against a 27-gauge needle with a 7.0 silk suture ligature. Sham-operated mice were conducted the similar procedure without ligation. 28 days after the TAC surgery, mice were euthanized by intraperitoneal (*i.p*.) administration of overdose pentobarbital sodium (150 mg/kg body weight), then the hearts were harvested for further examination.

For obesity-induced cardiac hypertrophy model, 6–8 weeks mice were randomly assigned and subjected to a 60% high fat diet (HFD) (Trophic Animal Feed High-Tech Co., Ltd, China) for 12 weeks to establish HFD-induced obesity. The mice were finally euthanized after 12 weeks by intraperitoneal (*i.p*.) administration of overdose pentobarbital sodium (150 mg/kg body weight), then the hearts were harvested for further examination.

### Cell culture and treatments

The primary NRCMs were isolated from Sprague-Dawley (SD) rats (1–2-day-old). Briefly, the cardiac tissues were promptly collected and minced into pieces, then repeatedly digested at 37 °C water bath for 5 min per cycle with 0.25% trypsin (Beyotime Biotechnology, China). Then the isolated cell suspension was filtered and centrifuged after 8–10 digestion cycles, and the collected cells were cultured at 37 °C with 5% CO2 for 1.5 h. Then cardiac fibroblasts were removed by a differential attachment technique. The collected NRCMs suspension were re-seeded into 6-well culture plates and stabilized in complete medium (DMEM + 10% fetal bovine serum (FBS) + 1% penicillin-streptomycin) and 1% 5-bromodeoxyuridine (BrdU, to inhibit the cardiac fibroblasts proliferation) for 2 days before various processing. H9C2 cells, a rat myocardial cell line, were obtained from Procell Life Science & Technology Co., Ltd. (Wuhan, China) and cultured in DMEM medium supplemented with 10% (v/v) FBS and 1% (v/v) penicillin-streptomycin. The H9C2 cells used in this study were free of mycoplasma contamination and were validated using the short tandem repeat (STR) method. The following cardiomyocyte hypertrophy model was established by treating NRCMs or H9C2 cells with phenylephrine (PE, 100 µM) or ISO (30 µM) for 48 h.

### Small interfering RNA (siRNA) transfection

For gene silencing, the rat ALKBH5 siRNA (si*Alkbh5*) and control siRNA (NC) (5′-UUCUCCGAACGUGUCACGUTT-3′) were obtained from GenePharma (Shanghai, China). NRCMs or H9C2 cells were transfected with RNA-lipid complexes containing siRNAs, lipofectamine RNAiMAX (Invitrogen) and opti-MEM (Gibco) according to the manufacturer’s instruction. The medium was replaced 12 h after transfection, and cardiomyocytes were cultured for another 24 h before being taken for further examination. The sequence of three ALKBH5 siRNA are as followed: si*Alkbh5*-1 (sense 5′- CUGCGCAACAAGUACUUCUTT-3′, antisense 5′-AGAAGUACUUGUUGCGCAGTT-3′), si*Alkbh5*-2 (sense 5′- GUGUCCGUGUCUUUCUUUATT-3′, antisense 5′- UAAAGAAAGACACGGACACTT-3′), si*Alkbh5*-3 (sense 5′-GCACCACGAUUGGAAACAATT-3′, antisense 5′- UUGUUUCCAAUCGUGGUGCTT-3′)

### Plasma construction and transfection

The construction of the *Alkbh5* overexpressing plasmid (*Alkbh5* OE) were performed by cloning the full length of the rat ALKBH5 cDNA into the pLV3-CMV-3×FLAG-EF1a-CopGFP-Puro lentiviral vector (MiaoLingBio, China). The empty lentiviral vector was named as the negative control. Sanger sequencing was used to verify these plasmids.

For plasma transfection, H9C2 cells or NRCMs were transfected with the above plasmid using the transfection reagent Lipofectamine 2000 (Invitrogen, USA) according to the manufacturer’s manual. the mixture was removed after 4 h and finally the gene overexpression efficiency was verified by immunoblot analyses.

### Immunofluorescence staining

The rationale of immunofluorescence staining analysis is to use fluorescently labeled antibodies as probes to localize and characterize specific antigens in tissues or cells. For the cellular immunofluorescence staining, cardiomyocytes were seeded in the glass-bottomed 24-well culture plates. After treatment with different stimulants, the cells were fixed with 4% paraformaldehyde, permeabilized with 0.25% Triton X-100 in PBS for 15 min followed by washing three times for 5 min with 1 × PBS. After blocking with 5% BSA for 30 min, cardiomyocytes were incubated with the appropriate primary antibody at 4 °C overnight. The primary antibodies used cellular immunofluorescence staining were as followed: ANP (Santa Cruz Biotechnology, sc-18811), BNP (Santa Cruz Biotechnology, sc-18817), ALKBH5 (AVIVA, ARP33751-P050) and α-actinin (Santa Cruz Biotechnology, sc-166524). Then the cardiomyocytes were stained with the Fluor-conjugated secondary antibody (Thermo Fisher Scientific) and the nuclei were counter-labeled with DAPI (Beyotime Biotechnology, C1005). Images of the stained cardiomyocytes were acquired using a fluorescence microscope (Carl-Zeiss-Promenade 10). The Image J software was used for the analysis of the cardiomyocyte surface area.

### Western blotting

The cardiomyocytes were homogenized in radioimmunoprecipitation assay (RIPA) lysis buffer (Pierce, Rockford, IL, USA) containing 1% protease and phosphatase inhibitor cocktails (APExBIO, USA) on ice. After centrifugation at 12,000 *g* for 15 min at 4 °C, the concentration of the supernatant protein was determined using a bicinchoninic acid (BCA) kit (Thermo Fisher Scientific). Then 30 μg of the whole protein was applied for western blotting and seperated by SDS-PAGE gels, then transferred to nitrocellulose (NC) membranes (Millipore). After blocking with 5% non-fat milk, the protein bands were incubated with appropriate primary antibodies at 4 °C overnight and its corresponding horseradish peroxidase (HRP)-conjugated secondary antibodies (1:5000; Jackson ImmunoResearch Inc., USA) for 1.5 h at RT. Chemiluminescence was generated and detected by ChemiDoc+ (Bio-Rad Laboratories, Inc) and each protein level was quantified using Image J software.

### Extraction of cytosolic and nuclear fraction

The extraction of cytosolic and nuclear fraction was performed using the NE-PER Nuclear and Cytoplasmic Extraction Reagents (Thermo Scientific, catalog no. 78835) according to the manufacturer’s instructions. Briefly, H9C2 cells were transfected with siAlkbh5 and then treated with PE for 48 h before collection for cytosolic and nuclear fraction extraction assay. The cytosolic and nuclear proteins were used for western blots, respectively.

### Real-time quantitative polymerase chain reaction (RT-qPCR)

RT-qPCR involves reverse transcription of RNA into complementary DNA (cDNA) followed by quantitative PCR amplification of the cDNA, enabling precise quantification of gene expression levels. Specifically, total RNA of cardiomyocytes was extracted using TRIzol Reagent (TaKaRa Biotechnology, Dalian, China) and then reversed to cDNA using the PrimeScript 1st Strand cDNA Synthesis Kit (TaKaRa Biotechnology, Dalian, China). The expressions of target genes were quantified by qRT-PCR using the SYBR Premix EX Taq II (Yeasen Biotech Co., Ltd. China) and performed on an iCycler iQ system (Bio-Rad, Hercules, CA, USA). Relative gene expression was normalized to β-tubulin or GAPDH using the standard 2^−△△Ct^ quantification method. The primer sequences were listed in Table S1.

### RNA immunoprecipitation (RIP)-qPCR

The RIP-qPCR assay combines RNA immunoprecipitation with quantitative PCR to investigate RNA-protein interactions, enabling the identification and quantification of specific RNA bound to target proteins. Briefly, cells were washed with cold PBS followed by lysed in lysis buffer containing RNase inhibitor and a protease inhibitor cocktail. The lysates were incubated with anti‐ALKBH5 antibody (Proteintech, 16837-1-AP) with rotation at 4 °C overnight. Protein A/G magnetic beads were washed with RIP washing buffer (50 mM Tris‐HCl pH 7.4, 150 mM NaCl, 1 mM MgCl_2_, 0.05% NP-40), followed by were added into lysates with rotation at 4 °C for 4 h. Co‐precipitated RNAs were eluted with Trizol Reagent. The RNA samples precipitated were extracted, reverse transcribed, and subjected to RT‐qPCR analysis and normalized to the input.

### MeRIP-qPCR

MeRIP-qPCR assay utilizes immunoprecipitation to obtain RNA fragments enriched with m6A modifications, followed by quantitative PCR to measure their abundance, elucidating the distribution and regulation of m6A modifications in RNA transcripts. Total RNA was extracted by Trizol reagent. The specific anti-m6A antibody was applied for m6A immunoprecipitation. Anti-m6A antibody was pre-bound to Protein A/G magnetic beads in reaction buffer at room temperature for 30 min. Then, the anti-m6A antibody-bound magnetic beads were washed, added to RNA, and incubated at 4 °C with rotation overnight followed by washed with low salt reaction buffer and high salt reaction buffer. M6A-antibody-bound RNA was extracted using HiPure Liquid RNA Kits (Magen Biotechnology, Guangzhou, China) according to the manufacturer’s instructions. Real-time qPCR assays were carried out following m6A-IP to quantify the m6A methylation levels of certain target genes.

### Dual-luciferase reporter assay

The Dual-luciferase reporter assay utilizes two luciferase enzymes, typically firefly and Renilla, to measure the activity of a promoter region or regulatory element by quantifying the ratio of the luminescence signals respectively, providing insight into gene expression or promoter/enhancer activity. Specifically, H9C2 cells were seeded in 96-well plates, and cultured overnight. The pGL3-Empty, pGL3-Stat3-WT, and pGL3-Stat3-Mut plasmids were constructed and obtained from MiaoLingBio, China. The above plasmids together with Control vector or *Alkbh5* OE were co-transfected into H9C2 cells by Lipofectamine 2000 reagent (Invitrogen). Then the cells were harvested 48 h after transfection for luciferease detection. Analysis of the luciferase activity was performed using the Dual-Luciferase Reporter Assay System according to the manufacturer’s instructions (Beyotime Biotechnology, Cat# RG027, China) and normalized to control Renilla luciferase levels.

### Gene expression analysis

To compare the expression levels of ALKBH5 in normal, dilated cardiomyopathy, and ischemic cardiomyopathy, we utilized published data from GSE116250, including 14 non-failing donors, 37 dilated cardiomyopathy samples, and 13 ischemic cardiomyopathy samples. We excluded the maximum and minimum expression values, along with outliers defined by Q3 ± 1.5 * (Q3–Q1). Gene expression analysis of the remaining 58 samples was conducted using the T-test method after filtering.

### Statistical analysis

The data were analyzed using GraphPad Prism 9.0 (GraphPad Software Inc., San Diego, CA, US) and presented as mean ± SD. Comparisons among groups were analyzed by using one-way or two-way ANOVA with the Bonferroni’s post hoc test for multiple groups, and when comparing between two groups using unpaired Student’s *t* test. Probability values of <0.05 was set as statistically significant.

## Supplementary information

Supplementary Material

Original Data

## Data Availability

The datasets generated during and/or analyzed during the current study are available from the corresponding author upon reasonable request.
