# Supplementary material for: The functional role of m6A demethylase ALKBH5 in cardiomyocyte hypertrophy
Source: Cell Death Dis. 2024 Sep 18;15(9):683. doi: 10.1038/s41419-024-07053-2 (PMC11410975; doi:10.1038/s41419-024-07053-2)
Supplement: Supplementary file 1 — Supplementary Material [file 41419_2024_7053_MOESM1_ESM.docx]

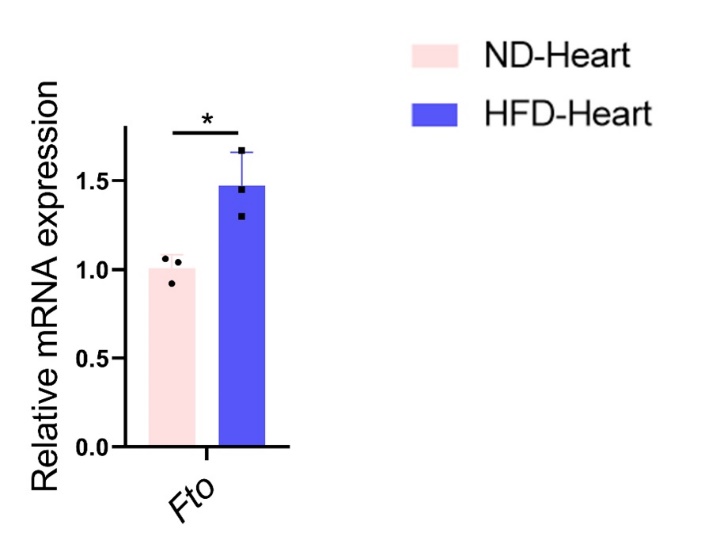


**Figure S1.** RT-qPCR assays were performed to evaluate the mRNA level of FTO (another enzyme that deletes m6A) in HFD-induced mice hearts. n=3, data shown are means ± SD. ∗*p* < 0.05.


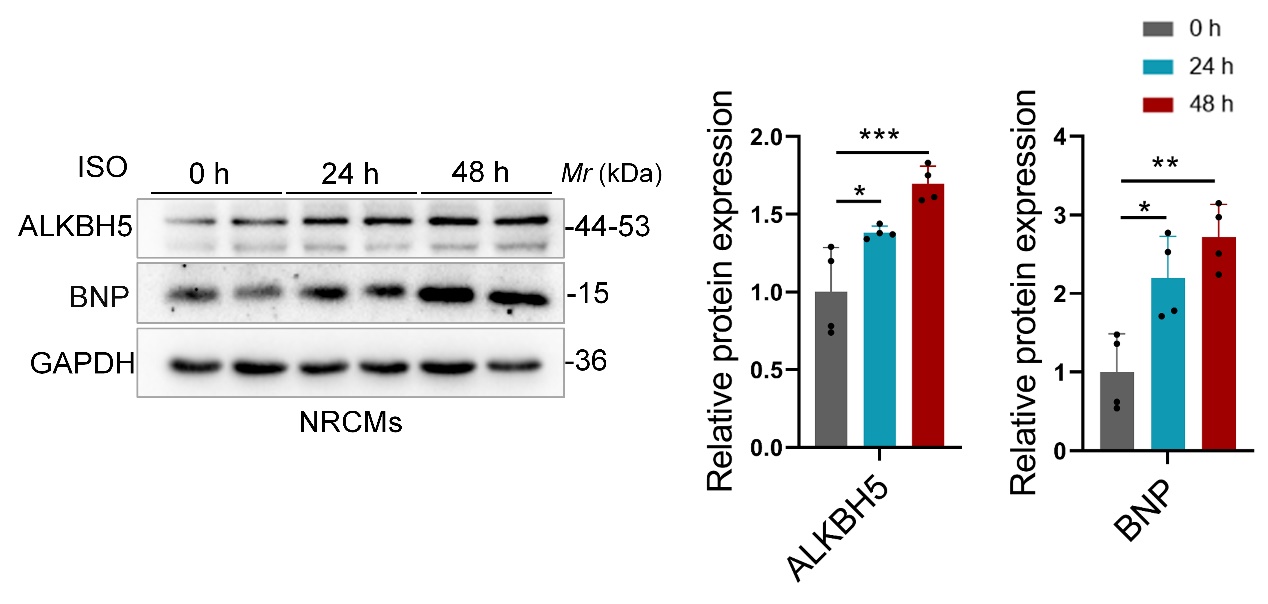


**Figure S2.** The protein expressions of ALKBH5 and BNP were examined in ISO (30 µM)-induced NRCMs by western blots. n=4, data shown are means ± SD. ∗*p* < 0.05, ∗∗*p* < 0.01, ∗∗∗*p* < 0.001.


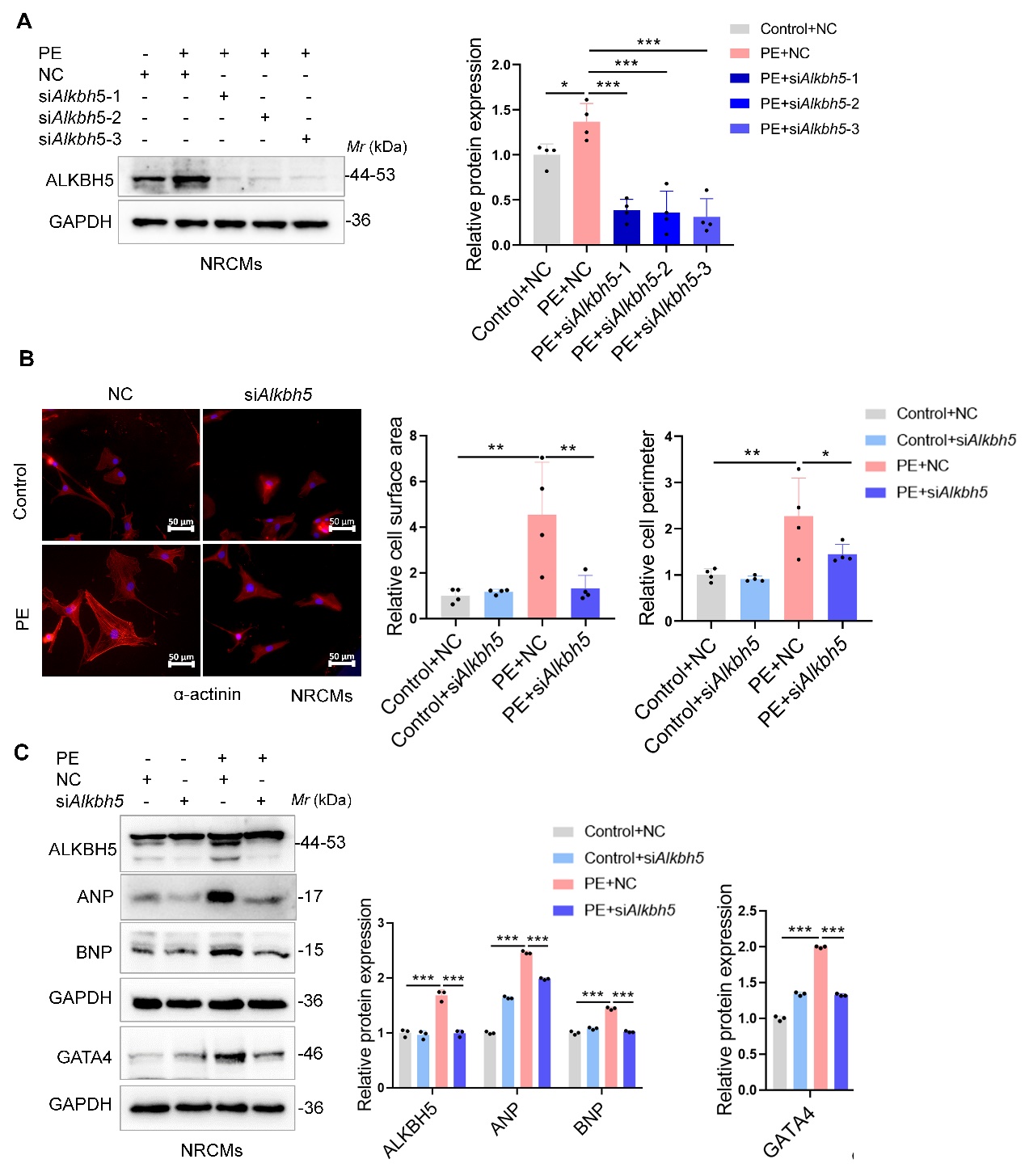


**Figure S3. ALKBH5 silence alleviates PE-induced neonatal rat cardiomyocytes hypertrophy*.*** NRCMs were transfected with si*Alkbh5* or NC and then treated with PE (100 µM) for 48 h. (**A**) The depletion efficiency of three si*Alkbh5*s were tested by western blots in NRCMs and the quantification was shown on the right. (**B**) Representative immunofluorescence images of α-actinin staining. Scale bar, 50 μm. Quantification of the relative cell surface area and cell perimeter were shown on the right. (**C**) Representative western blots (left) and quantification (right) of ALKBH5, ANP, BNP, and GATA4 proteins. n=3 per group, data shown are means ± SD. ∗*p* < 0.05, ∗∗*p* < 0.01, ∗∗∗*p* < 0.001.


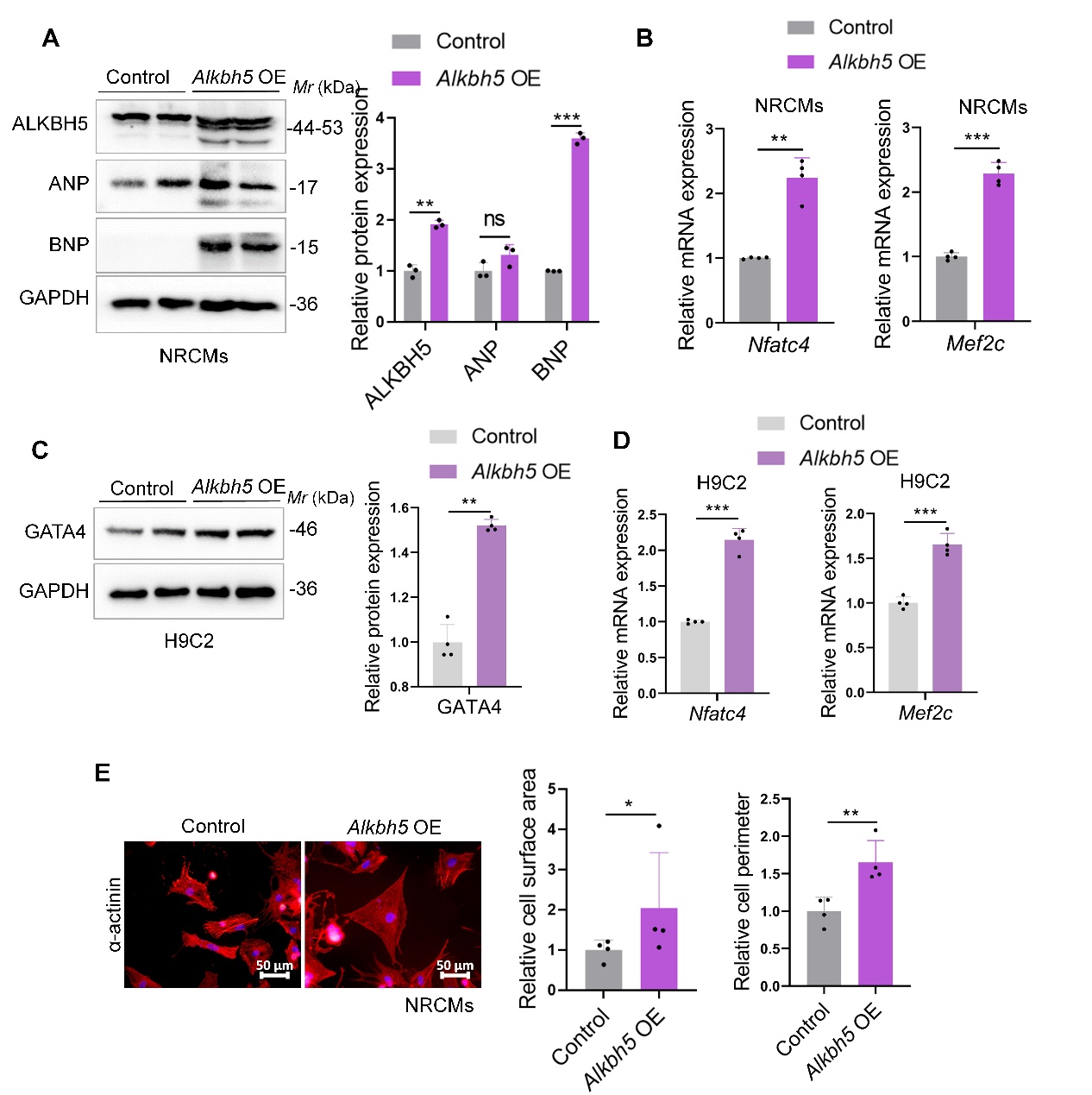


**Figure S4. ALKBH5 overexpression promotes neonatal rat cardiomyocytes hypertrophy.** (**A**) Representative western blots (left) and quantification (right) of ALKBH5, ANP, and BNP in *Alkbh5* OE-treated NRCMs. (**B**) The mRNA level of *Nfatc4* and *Mef2c* gene in *Alkbh5* OE-induced NRCMs were detected by qRT-PCR. (**C**) Representative western blots (left) and quantification (right) of GATA4 in *Alkbh5* OE-treated H9C2 cells. (**D**) The mRNA level of *Nfatc4* and *Mef2c* gene in *Alkbh5* OE-treated H9C2 cells were detected by qRT-PCR. (**E**) Representative immunofluorescence images of α-actinin staining in *Alkbh5* OE-treated NRCMs. Quantification of the relative cell surface area and cell perimeter were shown on the right. Data are expressed as the mean ± SD. ∗*p* < 0.05, ∗∗*p* < 0.01, ∗∗∗*p* < 0.001.


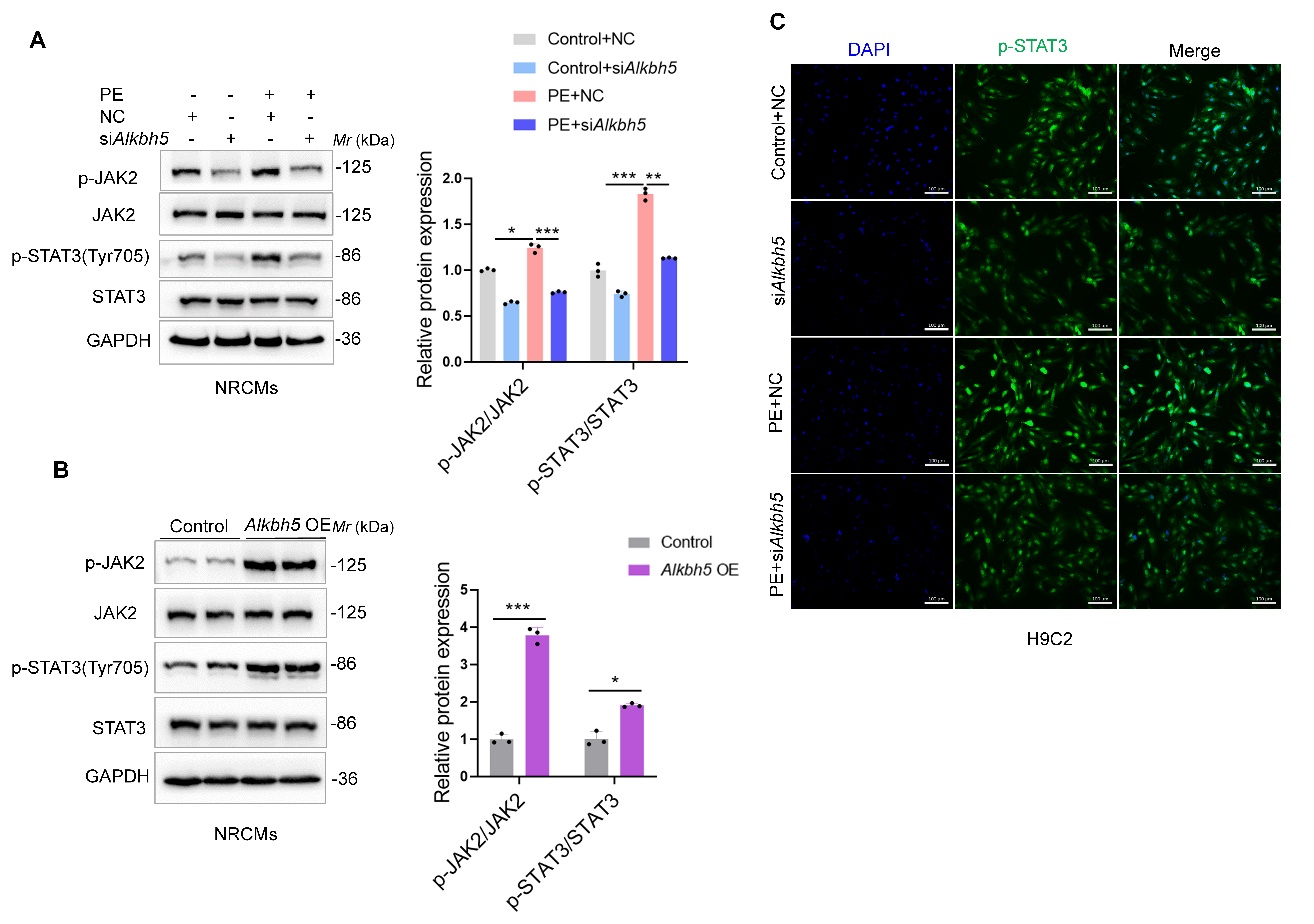


**Figure S5. ALKBH5 modulates STAT3 phosphorylation in cardiomyocyte hypertrophy.** (**A-B**) NRCMs were transfected with *Alkbh5* OE alone or si*Alkbh5* upon PE treatment. Immunoblot analysis was performed to detect the levels of p-JAK2 and p-STAT3(Y705), as well as the total protein expressions of JAK2 and STAT3. (**C**) The immunofluorescence staining was performed to detect the STAT3 location in H9C2 cells treated with si*Alkbh5* or NC and then stimulated with PE. Data are expressed as the mean ± SD. ∗*p* < 0.05, ∗∗*p* < 0.01, ∗∗∗*p* < 0.001.


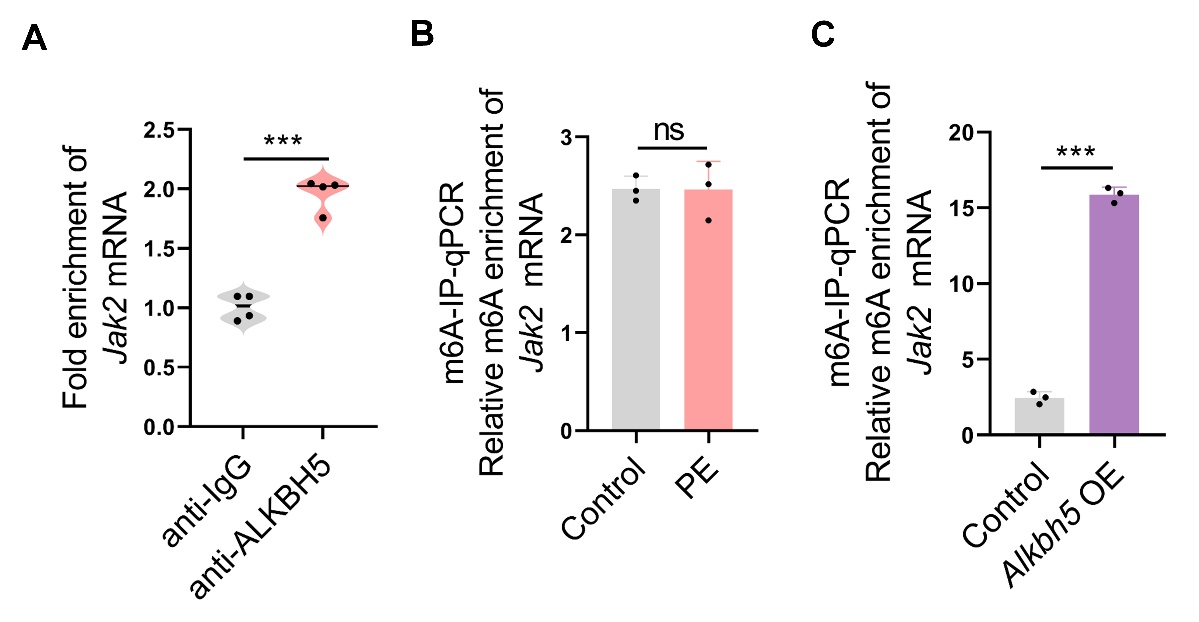


**Figure S6. ALKBH5 interacts with *Jak2* mRNA independent of m6A modification.** (**A**) Relative enrichment of *Jak2* mRNA associated with ALKBH5 protein was identified by RIP-qPCR assays using ALKBH5 antibody. n = 4. (**B**) MeRIP-qPCR assays were performed to detect the relative m6A enrichment of *Jak2* mRNA in PE-induced H9C2 cells. (**C**) MeRIP-qPCR assays were performed to detect the relative m6A enrichment of *Jak2* mRNA in *Alkbh5* OE-induced H9C2 cells. Data are expressed as the mean ± SD. ∗∗∗*p* < 0.001.

**Table S1** Primers used for RT-qPCR.

| Gene name | Primer sequence (5´ to 3´) |
| --- | --- |
| Rat-*Nppa*-F  Rat-*Nppa*-R | CAGGCCATATTGGAGCAAATC |
|  | CTCATCTTCTACCGGCATCTT |
| Rat-*Nppb*-F  Rat-*Nppb*-R | ACAGCTCTCAAAGGACCAAG  TGGAATTTCGAAGTCTCTCCTG |
| Rat-*Myh7*-F  Rat-*Myh7*-R | CCATCTCTGACAACGCCTATC  TCTTGGTGTTGACGGTCTTAC |
| Rat-*Alkbh5*-F  Rat-*Alkbh5*-R | CCACCAAACGGAAGTACCAG  TAGCCCTCGCCGAAGAAGTA |
| Rat-*tubulin*-F | GACCTGGAACCCACAGTTATT |
| Rat-*tubulin*-R | ATCTTCCTTGCCTGTGATGAG |
| Rat-*Gapdh*-F | ACTCTACCCACGGCAAGTTC |
| Rat-*Gapdh*-R | TGGGTTTCCCGTTGATGACC |
| Mouse-*Alkbh5*-F  Mouse-*Alkbh5*-R | TGCGCTGAAACCCAAAAGGT  GCAATCTTCCGAGGACTCATAG |
| Mouse-*Gapdh*-F  Mouse-*Gapdh*-R | GTTTCCTCGTCCCGTAGACA GATGGGCTTCCCGTTGATGA |

**Table S2** Primers used for MeRIP-qPCR

| Gene name | Primer sequence (5´ to 3´) |
| --- | --- |
| Rat-*Stat3*-3‘UTR-F  Rat-*Stat3*-3‘UTR-R  Rat-*Stat3* CDS-1318-F  Rat- *Stat3* CDS-1318-R  Rat-*Stat3* 3’-UTR-2953-F  Rat-*Stat3* 3’-UTR-2953-R  Rat-*Stat3* 3’-UTR-3153-F  Rat- *Stat3* 3’-UTR-3153-R  Rat-*Jak2*-3‘UTR -F  Rat-*Jak2*-3‘UTR -R | TTGTGTCGAGAGGGATTGCC |
|  | AACGGGGAGCATACAATCGG  ACAAAAGTCAGGTTGCTGGTC  TTTGTGTTCGTGCCCAGAATG  GAGAACTGAGTGAGCGTGGG  ATCAGCTCACAGAAAGGGGC  CGGCTCTGCACTTTCAACCT  CCGAAAAGGCTGTTAACCGAG  GTCCACGGGCCCTTATCAT  ACGCTTCCAAAGTCTTGAGCA |
