# Supplementary material for: The functional role of m6A demethylase ALKBH5 in cardiomyocyte hypertrophy
Source: Cell Death Dis. 2024 Sep 18;15(9):683. doi: 10.1038/s41419-024-07053-2 (PMC11410975; doi:10.1038/s41419-024-07053-2)
Supplement: Supplementary file 2 — Original Data [file 41419_2024_7053_MOESM2_ESM.pdf]

Figure 1

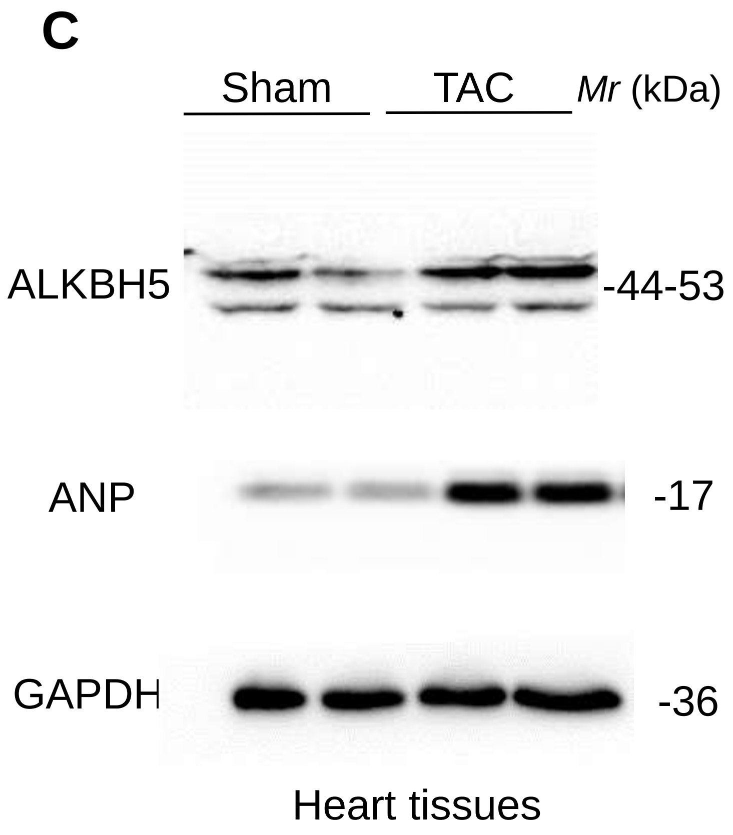
C
Sham
TAC
Mr (kDa)
ALKBH5
-44-53
ANP
-17
GAPDH
-36
Heart tissues

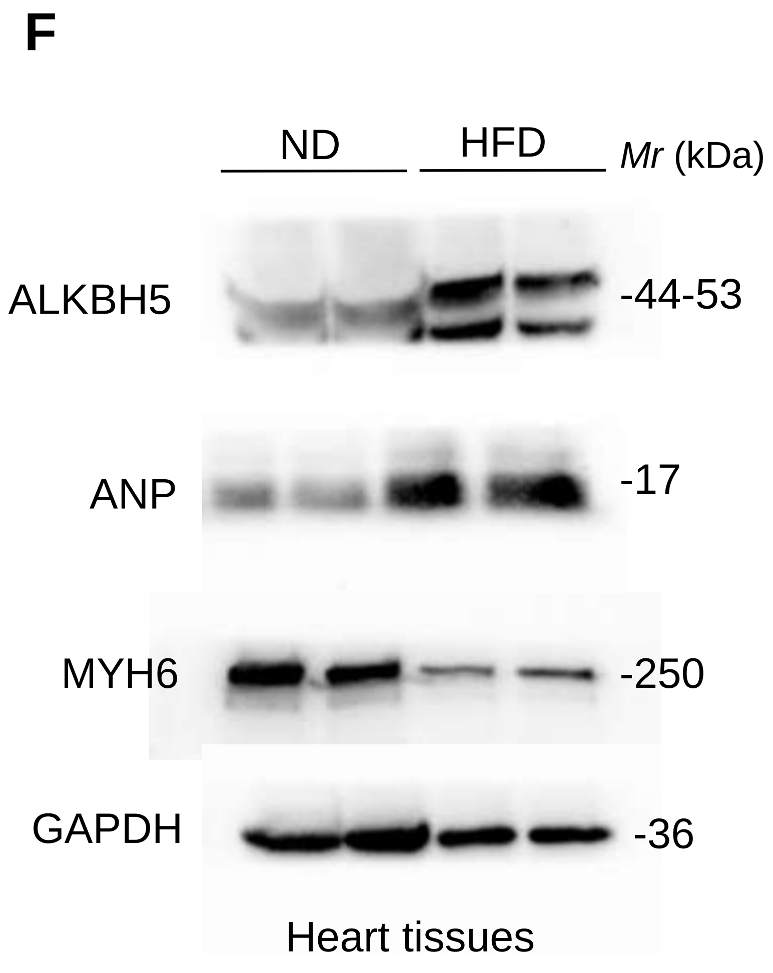
F
ND
HFD
Mr (kDa)
ALKBH5
-44-53
ANP
-17
MYH6
-250
GAPDH
-36
Heart tissues

Figure 2

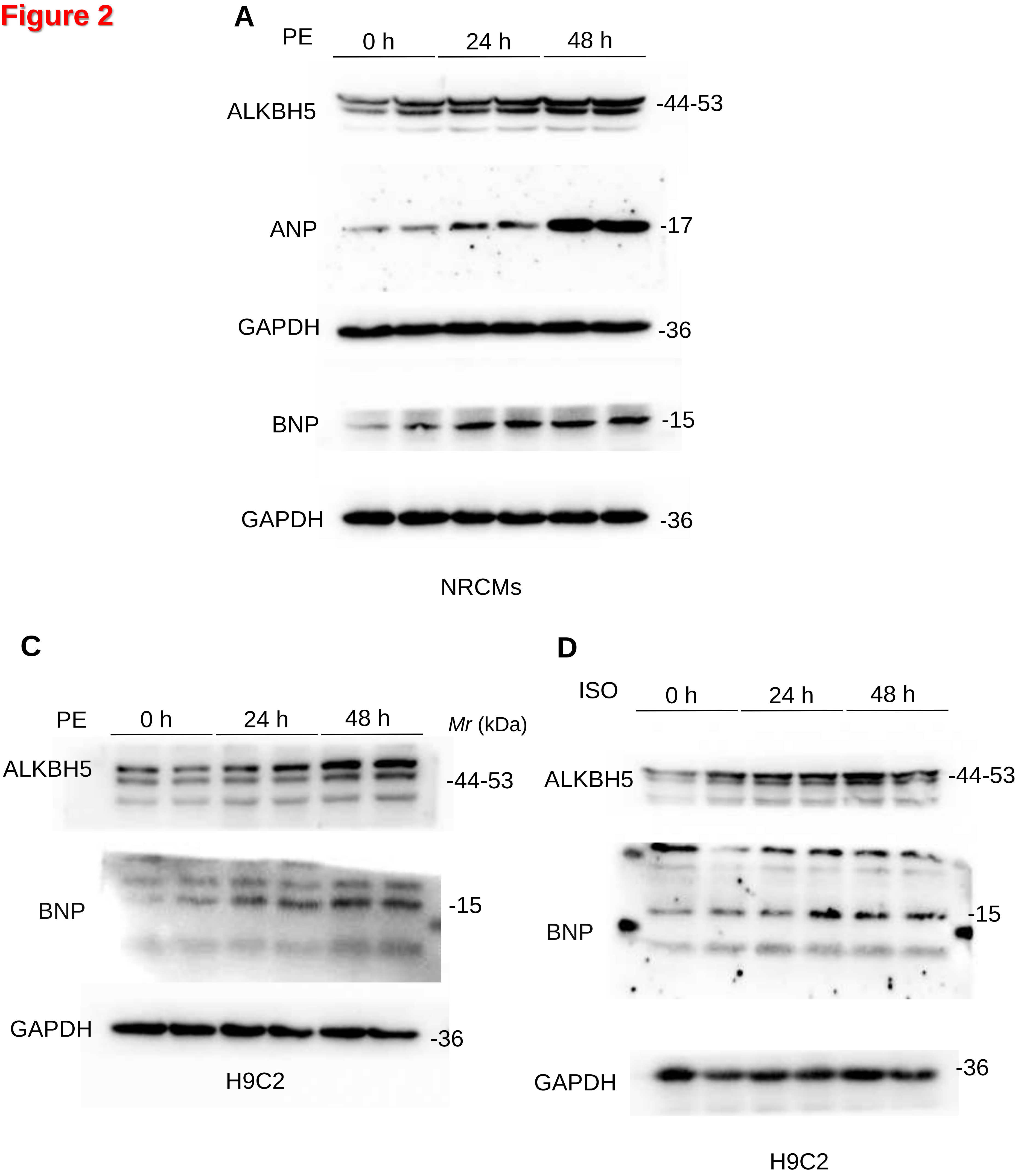
A
PE
0 h
24 h
48 h
ALKBH5
-44-53
ANP
-17
GAPDH
-36
BNP
-15
GAPDH
-36
NRCMs
C
PE
0 h
24 h
48 h
Mr (kDa)
ALKBH5
-44-53
BNP
-15
GAPDH
-36
H9C2
D
ISO
0 h
24 h
48 h
ALKBH5
-44-53
BNP
-15
GAPDH
-36
H9C2

Figure 3

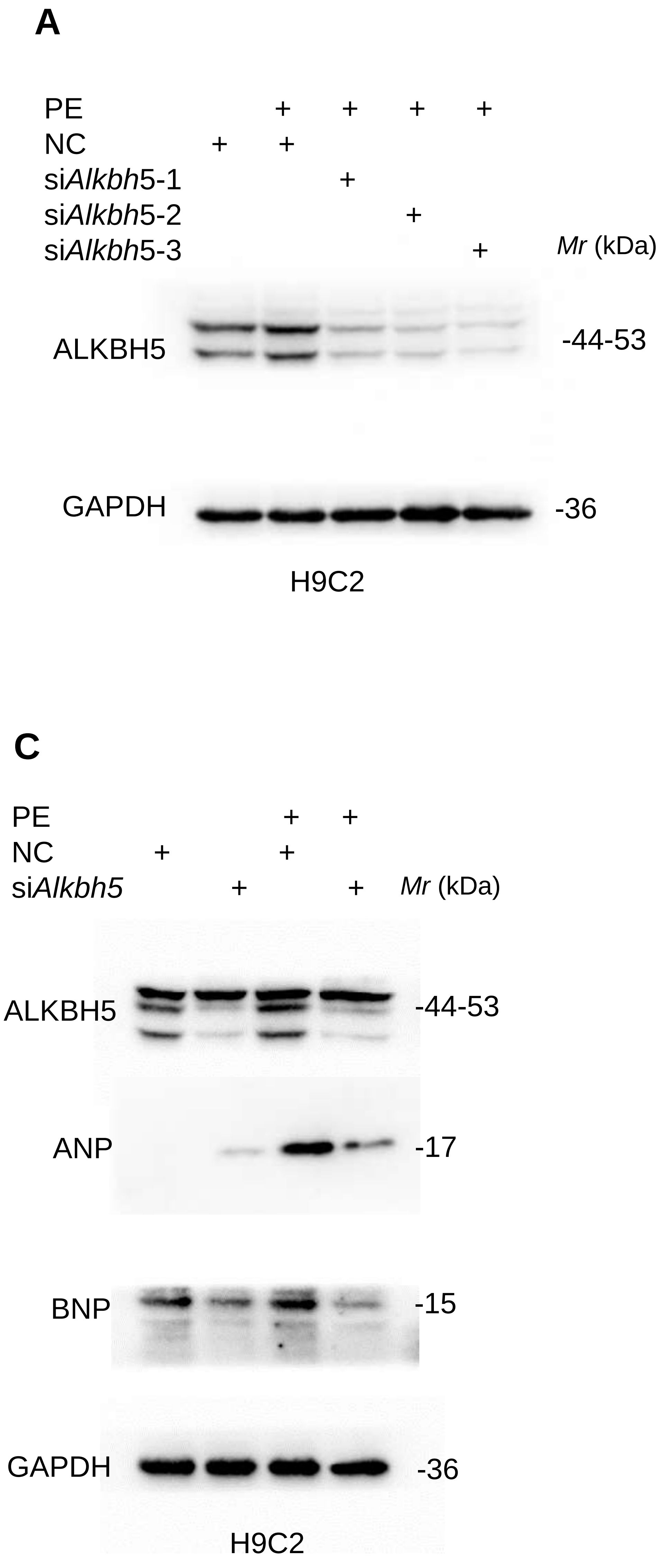
A
PE
NC
siAlkbh5-1
siAlkbh5-2
siAlkbh5-3
Mr (kDa)
ALKBH5
-44-53
GAPDH
-36
H9C2
C
PE
NC
siAlkbh5
Mr (kDa)
ALKBH5
-44-53
ANP
-17
BNP
-15
GAPDH
-36
H9C2

Figure 4

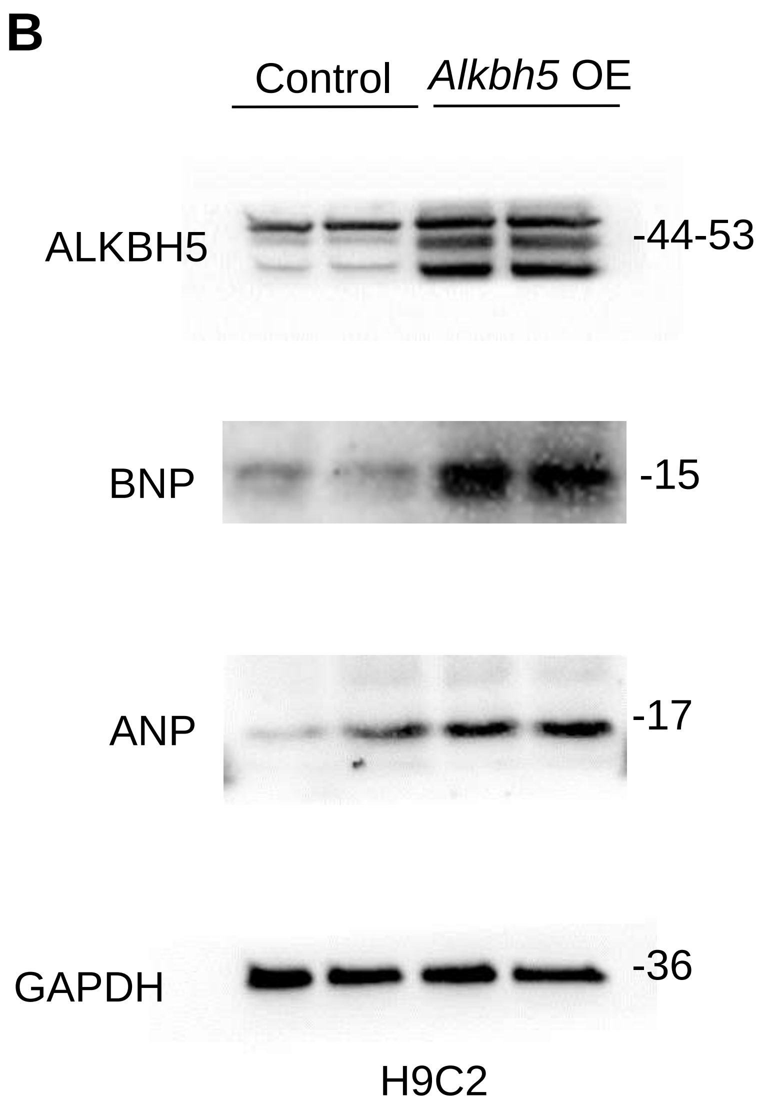

Figure 5

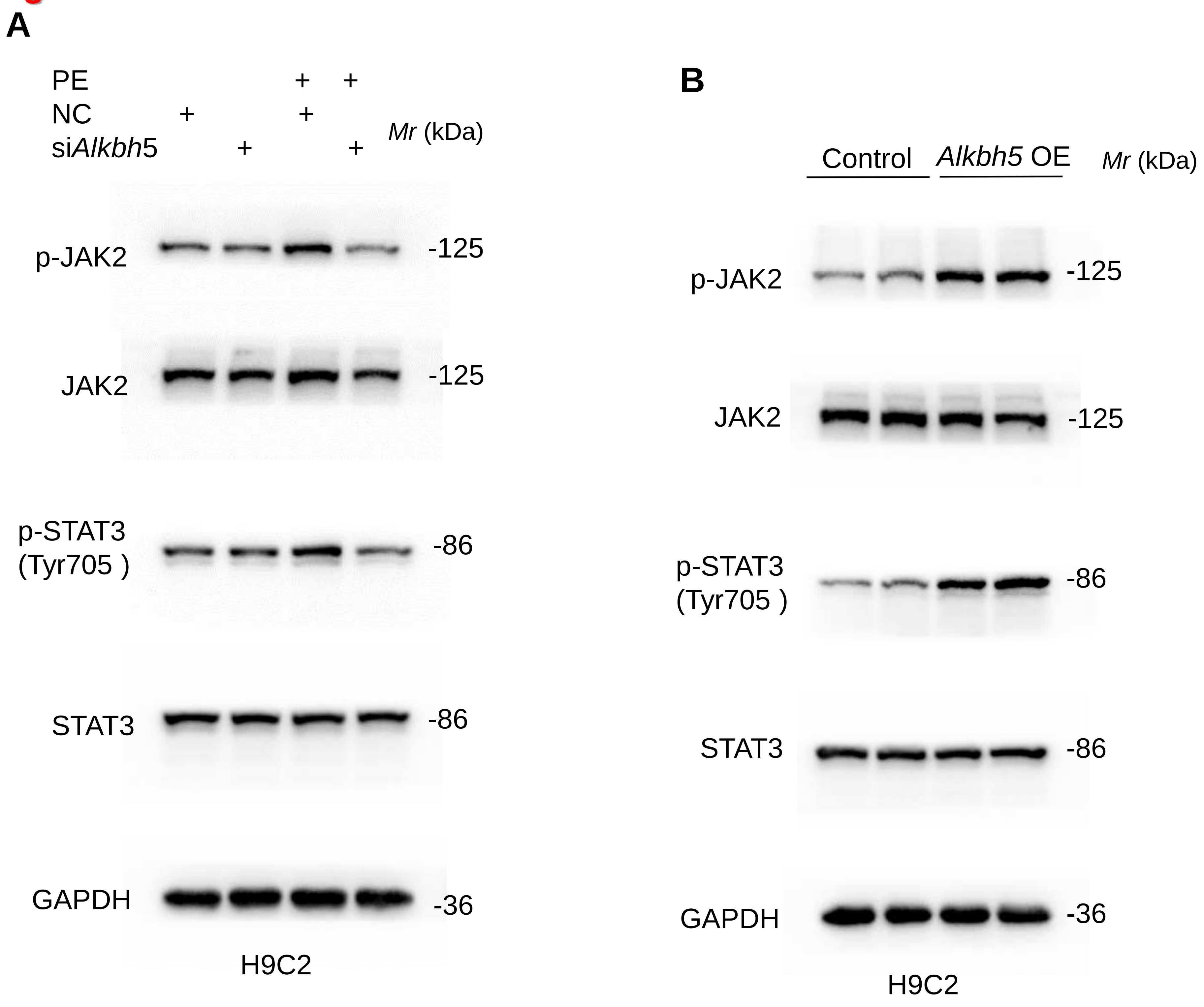
A
PE + +
NC + +
siAlkbh5 + +
Mr (kDa)
p-JAK2 -125
JAK2 -125
p-STAT3 (Tyr705) -86
STAT3 -86
GAPDH -36
H9C2
B
Control
Alkbh5 OE
Mr (kDa)
p-JAK2 -125
JAK2 -125
p-STAT3 (Tyr705) -86
STAT3 -86
GAPDH -36
H9C2

Figure 5

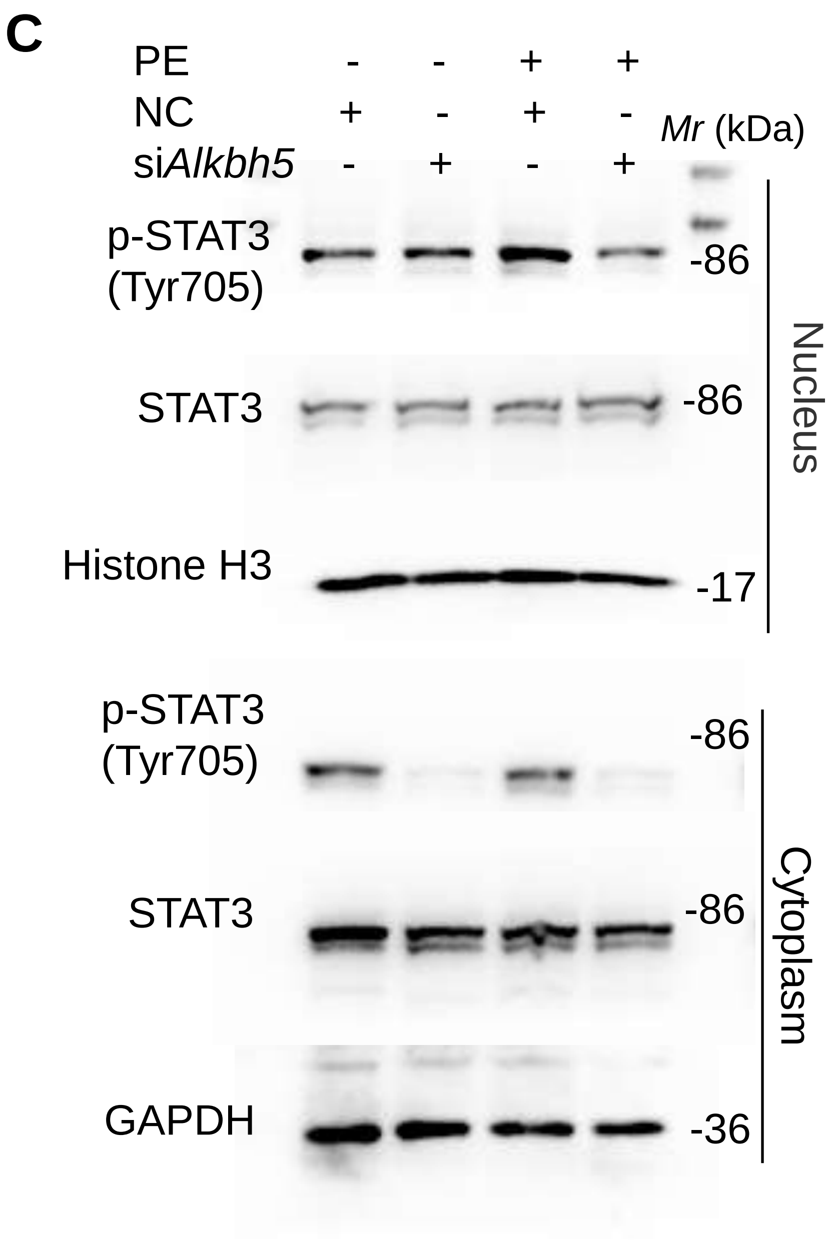
C
PE - - + +
NC + - + -
siAlkbh5 - + - +
Mr (kDa)
p-STAT3 (Tyr705)
-86
STAT3
-86
Histone H3
-17
Nucleus
p-STAT3 (Tyr705)
-86
STAT3
-86
GAPDH
-36
Cytoplasm

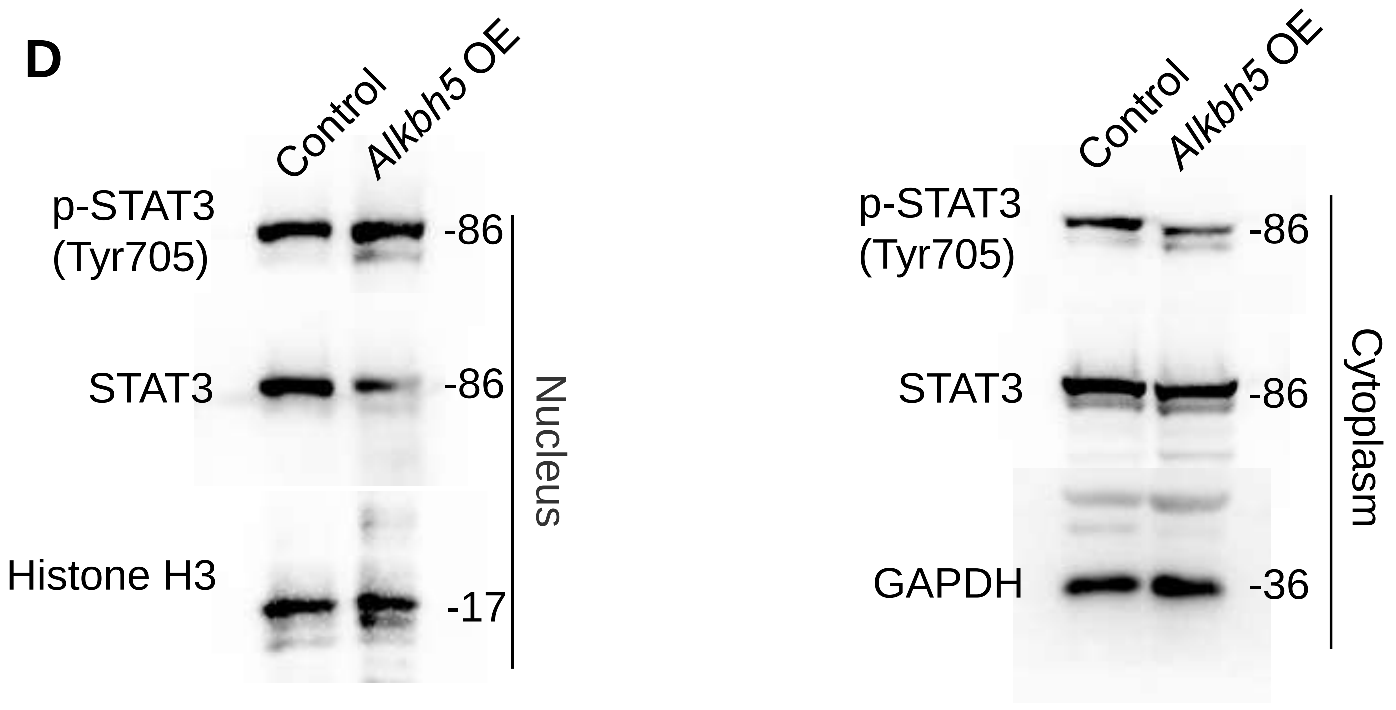
D
Control
Alkbh5 OE
p-STAT3 (Tyr705)
-86
STAT3
-86
Histone H3
-17
Nucleus
Control
Alkbh5 OE
p-STAT3 (Tyr705)
-86
STAT3
-86
GAPDH
-36
Cytoplasm

Figure 5

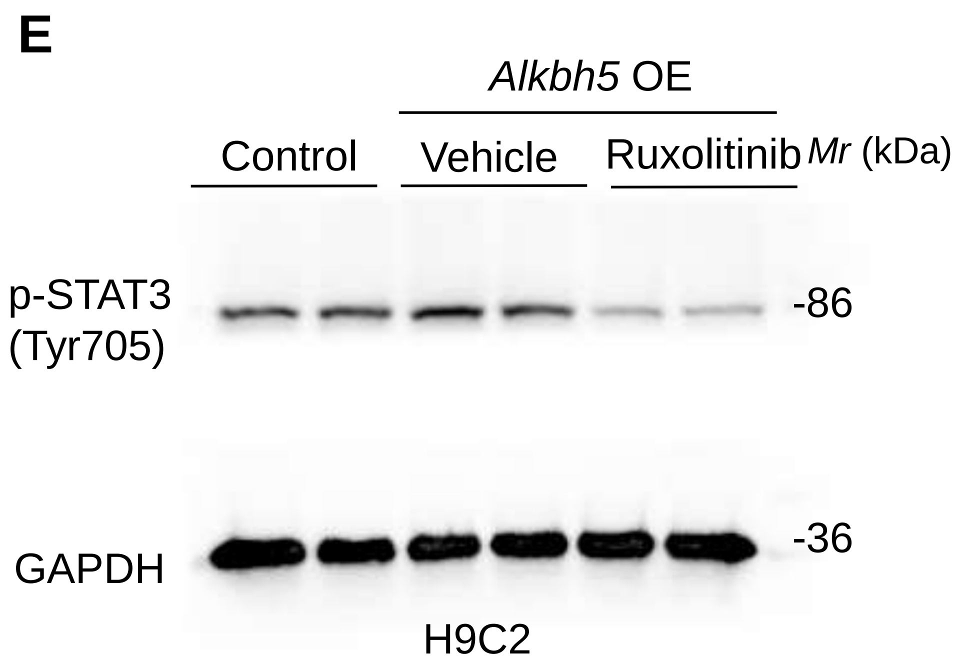
E
Alkbh5 OE
Control
Vehicle
Ruxolitinib
Mr (kDa)
p-STAT3
(Tyr705)
-86
GAPDH
-36
H9C2

Figure 7

A

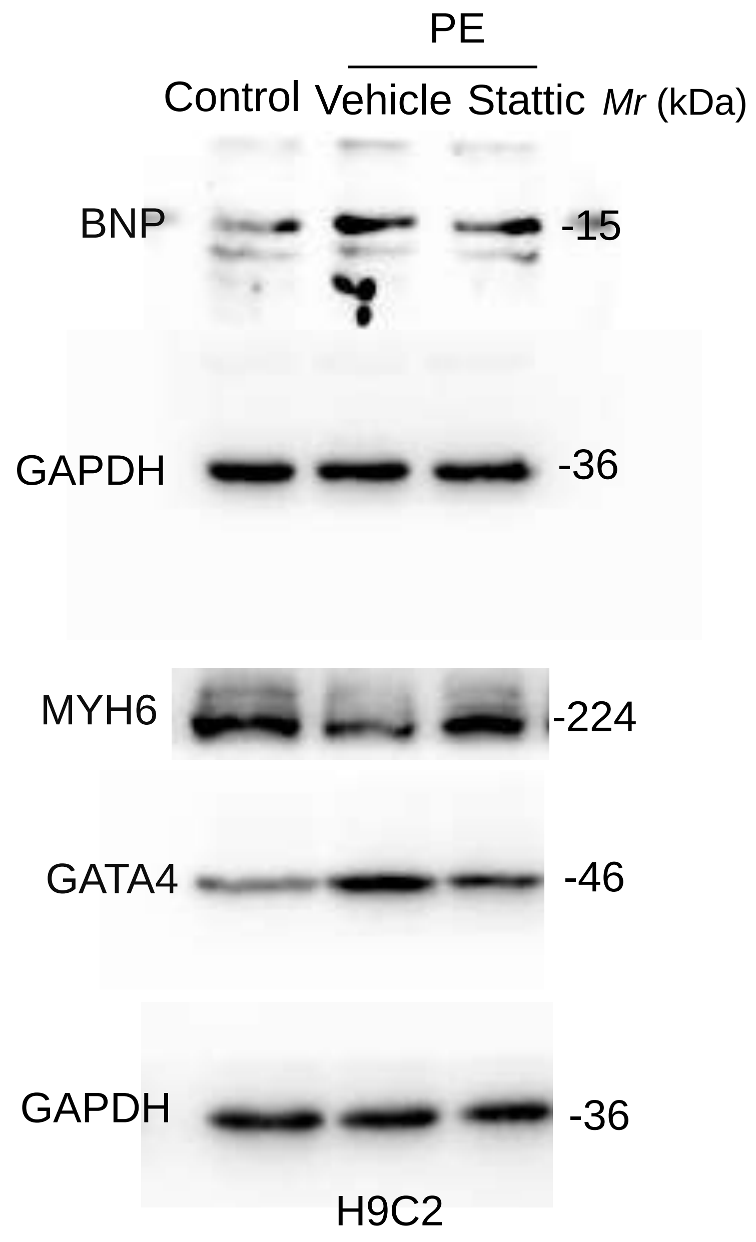
PE
Control
Vehicle
Stattic
Mr (kDa)
BNP
-15
GAPDH
-36
MYH6
-224
GATA4
-46
GAPDH
-36
H9C2

C

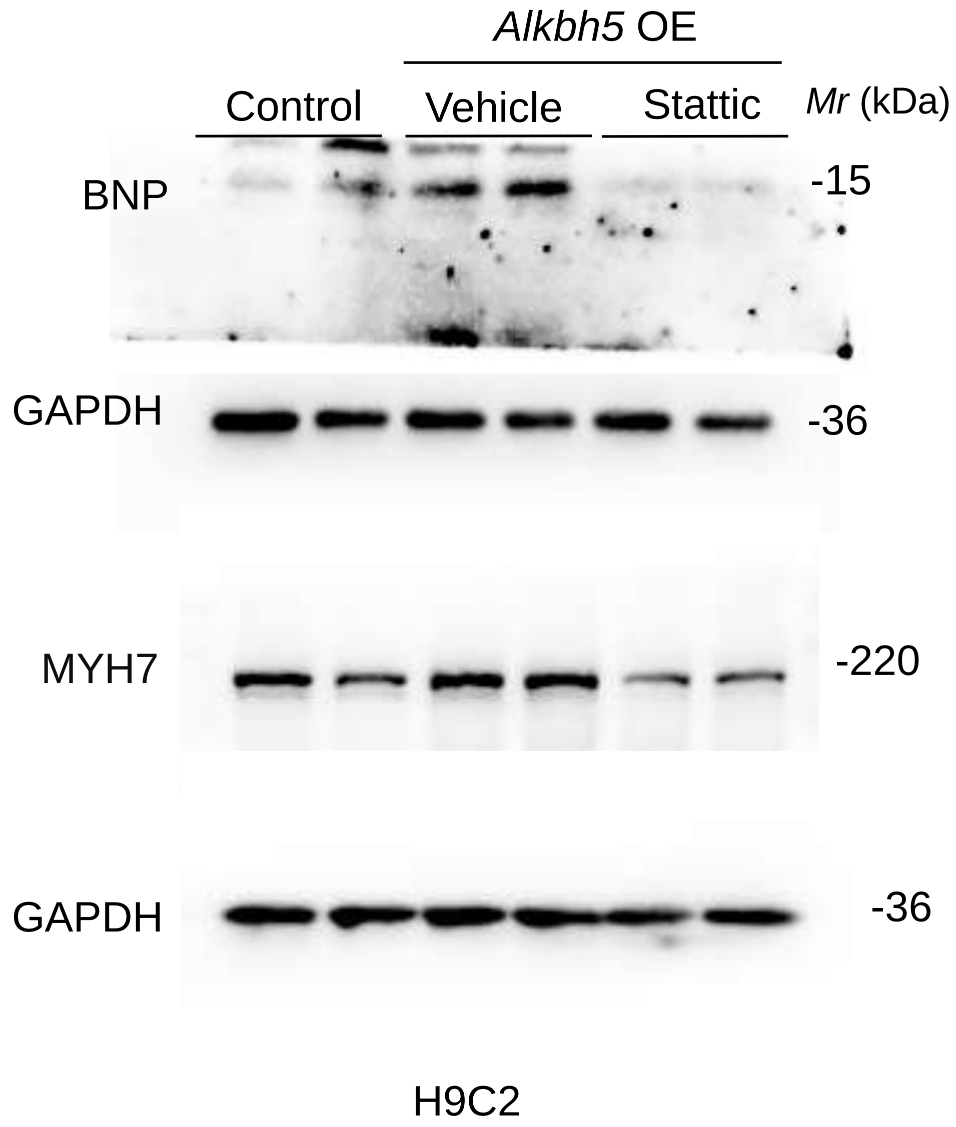
Alkbh5 OE
Control
Vehicle
Stattic
Mr (kDa)
BNP
-15
GAPDH
-36
MYH7
-220
GAPDH
-36
H9C2

Figure S2

B

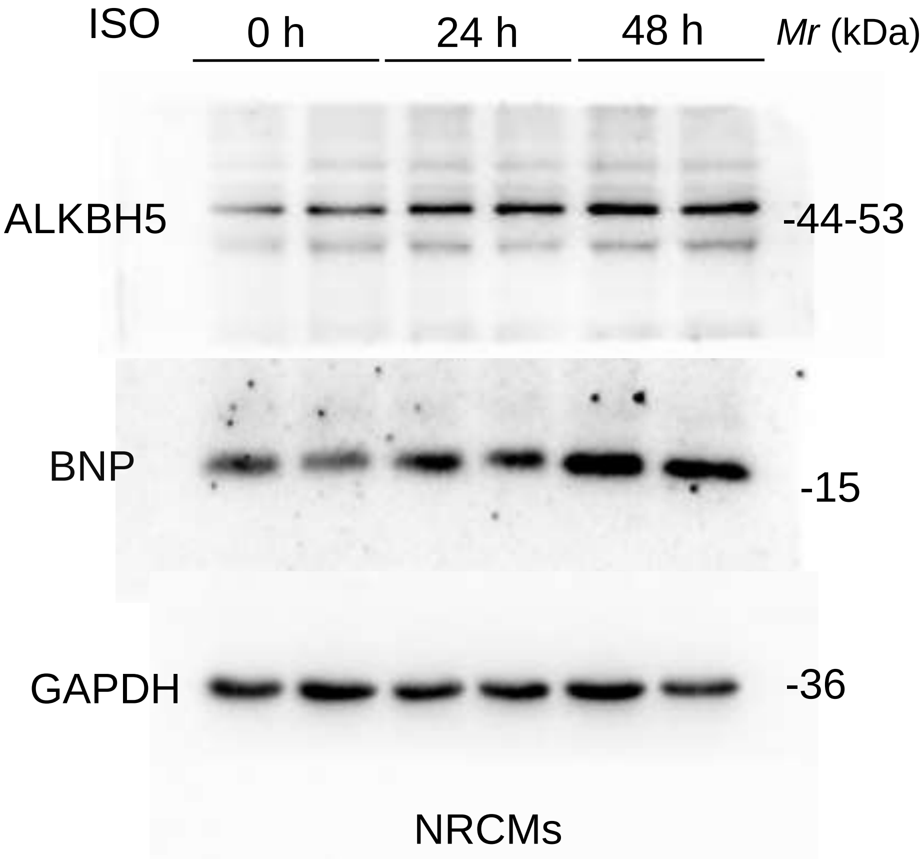

Figure S3

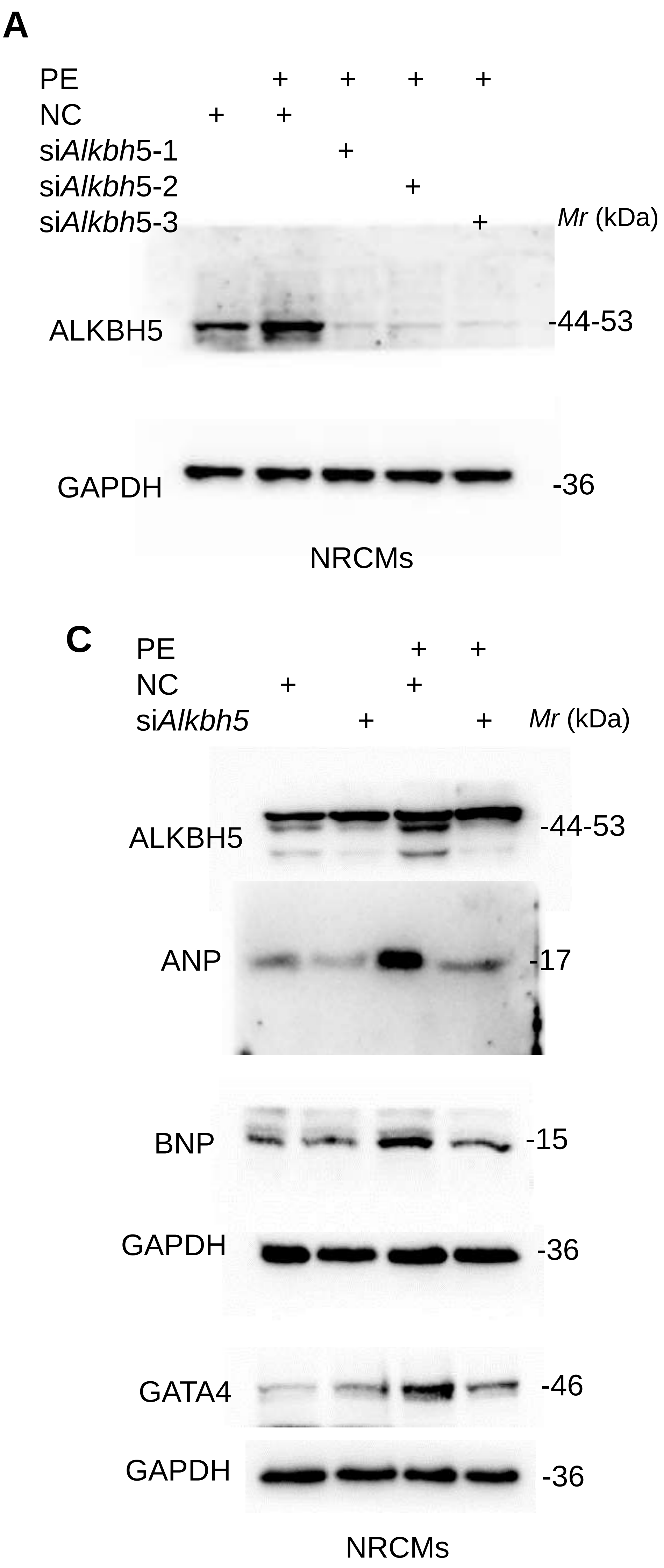
A
PE
NC
siAlkbh5-1
siAlkbh5-2
siAlkbh5-3
Mr (kDa)
ALKBH5
-44-53
GAPDH
-36
NRCMs
C
PE
NC
siAlkbh5
Mr (kDa)
ALKBH5
-44-53
ANP
-17
BNP
-15
GAPDH
-36
GATA4
-46
GAPDH
-36
NRCMs

Figure S4

A

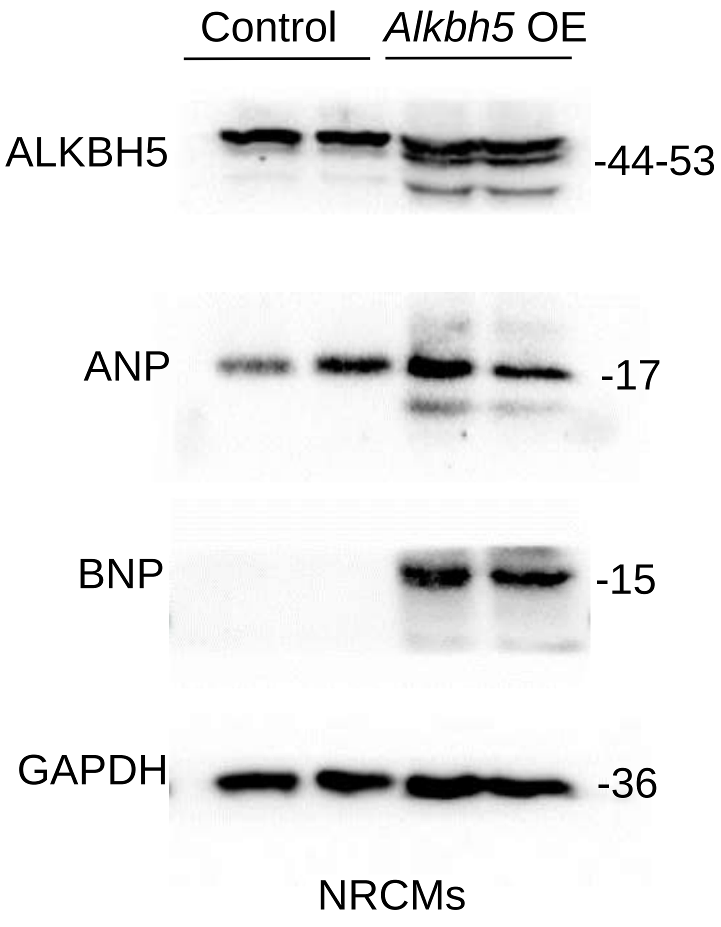

Control
Alkbh5 OE
ALKBH5
-44-53
ANP
-17
BNP
-15
GAPDH
-36
NRCMs

C

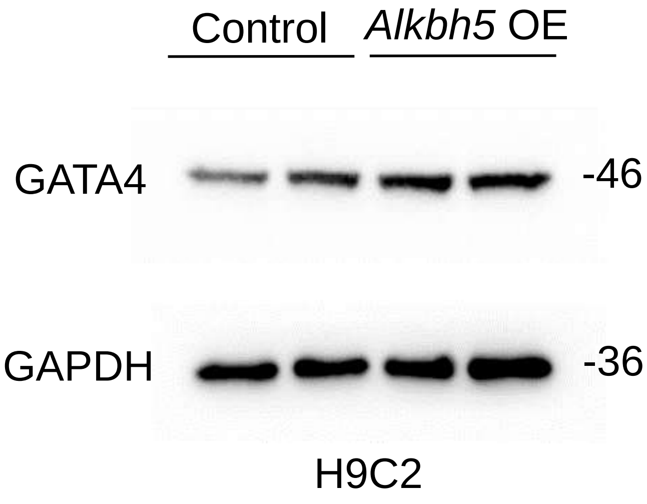

Control
Alkbh5 OE
GATA4
-46
GAPDH
-36
H9C2

Figure S4

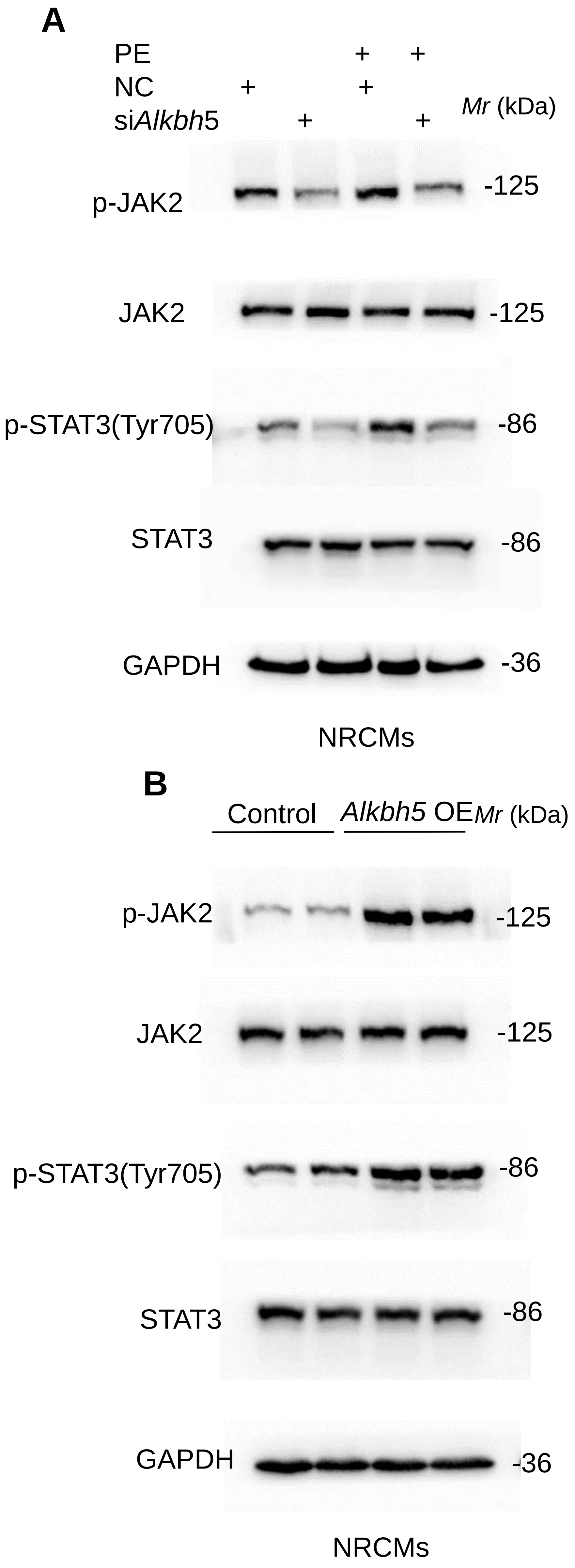
A
PE
NC
siAlkbh5
Mr (kDa)
p-JAK2
-125
JAK2
-125
p-STAT3(Tyr705)
-86
STAT3
-86
GAPDH
-36
NRCMs
B
Control
Alkbh5 OE
Mr (kDa)
p-JAK2
-125
JAK2
-125
p-STAT3(Tyr705)
-86
STAT3
-86
GAPDH
-36
NRCMs
